# Genome-Based Reclassification of Two *Haloarcula* Species and Characterization of *Haloarcula montana* sp. nov.

**DOI:** 10.3390/biology14060615

**Published:** 2025-05-27

**Authors:** Ji-Qiang Liu, Ling-Rui Zhu, Ya-Ling Mao, Xue Ma, Jing Hou

**Affiliations:** 1 State Key Laboratory of Submarine Geoscience, Second Institute of Oceanography, Ministry of Natural Resources, Hangzhou 310012, China; liujq@sio.org.cn; 2 School of Food and Biological Engineering, Jiangsu University, Zhenjiang 212013, China; 2212218050@stmail.ujs.edu.cn (L.-R.Z.); 92212218050@stmail.ujs.edu.cn (Y.-L.M.); 19599965169@163.com (X.M.)

**Keywords:** *Haloarcula*, novel halophilic archaeon, salt lake, comparative genomics, taxogenomics

## Abstract

The genus *Haloarcula* is one of the most extensively studied genera within the class *Halobacteria*. In this study, analyses of average nucleotide identity (ANI), average amino acid identity (AAI), digital DNA–DNA hybridization (dDDH) values, and phylogenomic data indicated that “*Haloarcula californiae*” ATCC 33799 and “*Haloarcula sinaiiensis*” ATCC 33800 served as reference strains for *Haloarcula marismortui*. Furthermore, a halophilic archaeal strain, GH36^T^, isolated from the inland Gahai Salt Lake in China, was subjected to comprehensive polyphasic taxonomic characterization. The ANI, AAI, and dDDH comparisons between strain GH36^T^ and other *Haloarcula* species were below the established thresholds for species demarcation. Strain GH36^T^ was assigned to a novel species within the genus *Haloarcula* based on phylogenetic, phylogenomic, and comparative genomic analyses. Moreover, distinct phenotypic differences were observed in strain GH36^T^. In strain GH36^T^, the primary phospholipids detected were phosphatidylglycerol (PG), phosphatidylglycerol phosphate methyl ester (PGP-Me), and phosphatidylglycerol sulfate (PGS), whereas the principal glycolipids were sulfated mannosyl glucosyl diether (S-DGD-1) and mannosyl glucosyl diether (DGD-1). Accordingly, the novel species *Haloarcula montana* sp. nov. is proposed to accommodate strain GH36^T^ (=CGMCC 1.62631^T^ = MCCC 4K00122^T^).

## Simple Summary

Taxogenomic analyses indicated that “*Haloarcula californiae*” ATCC 33799 and “*Haloarcula sinaiiensis*” ATCC 33800 served as reference strains for *Haloarcula marismortui*. Furthermore, a novel species within the genus *Haloarcula*, designated *Haloarcula montana* sp. nov., was proposed based on a comprehensive polyphasic characterization of the type strain GH36^T^, which was isolated from a salt lake in China.

## 1. Introduction

Archaea, as the third domain of life, are distinct from and stand alongside bacteria and eukarya, often thriving in extreme or specialized habitats such as high-salt, anaerobic, and high-temperature conditions [1]. Among these, halophilic archaea (class *Halobacteria*) are of great interest because of their unique physiological properties and their ability to adapt to high-salt conditions [2]. These organisms typically necessitate a minimum of 1.5 M NaCl for growth and are predominantly found in hypersaline ecosystems, including salt lakes, salt mines, saltpans, and salted foods [3,4,5]. They represent an invaluable resource for diverse industrial and biotechnological applications. Their unique biomolecules include salt-tolerant enzymes [6,7], light-driven proton pumps like bacteriorhodopsin, and sustainable biopolymers such as polyhydroxyalkanoates (PHAs) and exopolysaccharides [8]. Given this broad potential, the discovery and characterization of novel halophilic archaeal strains remain a compelling avenue for biotechnological innovation.

As of March 2025, the class *Halobacteria* comprises two orders, 10 families, 86 genera, and 410 species. Within this diverse group, the genus *Haloarcula* stands out as one of the most extensively studied, encompassing 26 species with validly published names and an additional 7 effectively published but not yet validated species (“*Haloarcula sediminis*”, “*Haloarcula brevis*”, “*Haloarcula regularis*”, “*Haloarcula sinaiiensis*”, “*Haloarcula rubripromontorii*”, “*Haloarcula taiwanensis*”, and “*Haloarcula californiae*”) (https://lpsn.dsmz.de/). Due to their physiological and ecological significance, *Haloarcula* species serve as key model organisms in haloarchaeal research. The taxonomic relationships between *Haloarcula* and *Halomicrocula* were previously ambiguous. Recent advances in comparative genomics and phylogenomic analyses have helped clarify their taxonomic relationships, leading to the reclassification of several species [9,10].

The genus *Haloarcula* was established with *Haloarcula vallismortis* as its type species, with the type strain first isolated from saline pools in Death Valley in 1978 [11]. Members of this genus have been recovered from diverse hypersaline habitats worldwide, including salt lakes, marine solar salterns, salt mines, and other saline ecosystems [9,10,12,13,14]. When cultured on agar plates, they typically form red-pigmented, moist, circular colonies. Most strains demonstrate optimal growth at neutral pH and moderate thermophily. Their NaCl requirements for growth vary significantly, reflecting adaptations to their native environments and specific osmotic regulation strategies. The polar lipid profile of *Haloarcula* species includes phosphatidylglycerol (PG), phosphatidylglycerol phosphate methyl ester (PGP-Me), phosphatidylglycerol sulfate (PGS), sulfated mannosyl glucosyl diether (S-DGD-1), mannosyl glucosyl diether (DGD-1), glucosyl mannosyl glucosyl diether (TGD-2), and other glycolipids.

In this study, whole-genome comparative analysis and phylogenomic investigation encompassing all currently recognized species of the genus *Haloarcula* were performed to elucidate the evolutionary relationships within this archaeal group. Concurrently, a novel halophilic archaeal strain, GH36^T^, isolated from the Gahai Salt Lake (Qinghai, China), was subjected to polyphasic taxonomic characterization.

## 2. Materials and Methods

### 2.1. Isolation and Cultivation of Halophilic Archaea

Strain GH36^T^ was isolated in 2020 from the sediment sample collected from Gahai Salt Lake (Qinghai Province, China; 37°11′52″ N, 96°52′7″ E; elevation: 2808 m). The sample exhibited moderate alkalinity (pH 7.2) and hypersalinity (18.5% total salts). The primary isolation was performed using neutral haloarchaeal medium (NHM) [15]. Sediment samples were homogenized and subjected to serial dilution (1:9, *w*/*v*) in sterile NHM broth before spread-plating onto NHM agar plates. Plates were incubated aerobically at 37 °C for a month until distinct colonies appeared. Pure cultures were obtained through ≥3 successive quadrant streaks on fresh NHM agar plates, which were subsequently cryopreserved at −20 °C in NHM broth supplemented with 15% (*v*/*v*) glycerol for long-term maintenance [16]. For comparative analyses, reference *Haloarcula* strains were cultured under identical conditions (NHM medium, 37 °C, aerobic incubation).

### 2.2. Phylogenetic Analysis Based on 16S rRNA and RNA Polymerase B Subunit (rpoB’) Genes

Genomic DNA extraction was performed using a commercial bacterial DNA isolation kit following the manufacturer’s protocol (CoWin Biosciences, Taizhou, China). Archaeal 16S rRNA genes were amplified via PCR employing the universal primer pair 20F (5′-ATTCCGGTTGATCCTGCCGG-3′) and 1452R (5′-AGGAGGTGATCCAGCCGCAG-3′), with subsequent cloning and sequencing performed as previously described [17]. Additionally, the *rpoB*’ gene sequences were retrieved from whole-genome data. Sequence similarity comparisons between the isolate and established *Haloarcula* species were determined using the EzBioCloud platform [18]. Phylogenetic reconstruction was conducted in MEGA 7 [19] utilizing the maximum-likelihood method [20]. Tree topologies were validated through 1000 bootstrap replicates to assess branch support, with *Halorutilus salinus* F3-133^T^ serving as the outgroup reference [21].

### 2.3. Genomic Characterization and Comparative Analysis

The whole-genome sequencing of strain GH36^T^ was conducted using PacBio Sequel technology, followed by comprehensive bioinformatic analyses. Overall genome-related indices (ORGIs) were calculated, and average nucleotide identity (ANI) calculations via the ANI calculator [22], digital DNA–DNA hybridization (dDDH) estimates using the Genome-to-Genome Distance Calculator [23], and average amino acid identity (AAI) assessments with the AAI calculator [24]. Phylogenomic placement was determined through Genome Taxonomy Database (GTDB)-based analysis with *Halobacterium bonnevillei* PCN9^T^ as the outgroup [25,26].

Orthologous clusters (OCs) were comparatively analyzed with reference strains using OrthoVenn3 (https://orthovenn3.bioinfotoolkits.net). Genome collinearity was analyzed using the Easyfig software (v2.2.5) [27]. Pangenome analysis was executed via the Integrative Pangenome Analysis (IPGA) platform [28]. Only genomes exhibiting >90% completeness and <5% contamination were included. The identity cutoff of all-vs-all BLAST and the frequency cutoff to define core genes were set as 70% and 0.95, respectively.

Functional annotation of the genome was performed through the Rapid Annotation using Subsystems Technology (RAST) server [29] that provided subsystem-based annotation, as well as the Kyoto Encyclopedia of Genes and Genomes (KEGG) database [30] that enabled metabolic pathway reconstruction. Potential biotechnologically relevant genes were identified through BlastP analysis (https://blast.ncbi.nlm.nih.gov/).

### 2.4. Phenotypic Determination

The phenotypic characterization of strain GH36^T^ was conducted in accordance with the updated minimal standards for taxonomic description of the class *Halobacteria* [31]. Cell morphology and motility were examined by phase-contrast microscopy (1000×) and scanning electron microscopy [32]. Gram staining followed the halophilic archaeal-adapted protocol [33]. Growth parameters, including NaCl/MgCl_2_ requirements, temperature range, and pH range, were determined using established methods [34]. Hydrolytic activity against casein, gelatin, starch, and Tween 80 was tested through hydrolysis zones on supplemented NHM agar plates. The antibiotic sensitivity of the strain was determined through distinct inhibition zones on NHM agar plates [35]. Polar lipids were analyzed through one- and two-dimensional thin-layer chromatography (TLC) [36].

## 3. Results and Discussion

### 3.1. Phylogenetic Analysis Based on 16S rRNA and rpoB’ Genes

Strain GH36^T^ harbored two copies of the 16S rRNA gene, designated *rrnA* (1474 bp) and *rrnB* (1473 bp), respectively, with 93.2% sequence similarity between them (Table S1). Comparative analysis revealed that the 16S rRNA gene sequences of GH36^T^ shared 89.5–99.9% similarity with currently recognized *Haloarcula* species (Table S1). The 16S rRNA gene sequence similarity threshold of 98.65% was proposed for prokaryotic species delineation [37]. However, several 16S rRNA gene sequence similarity values between *Haloarcula* species exceeded this threshold (Table S1). These findings underscored the limitations of relying solely on 16S rRNA gene comparisons for definitive taxonomic classification. Additional genome-based comprehensive analyses are essential for accurate species discrimination within this genus.

Phylogenetic reconstruction based on 16S rRNA gene sequences positioned *rrnA* and *rrnB* of GH36^T^ within a well-supported clade (99% bootstrap) alongside *Haloarcula halophila* DFY41^T^, further grouping within a larger cluster containing *Haloarcula pelagica* YJ-61-S^T^ (Figure S1a).

The *rpoB’* gene (1827 bp) of GH36^T^ exhibited 88.3–97.6% sequence similarity with other *Haloarcula* species, with the highest similarity observed with *Haloarcula halophila* DFY41^T^ (Table S2). Phylogenetic analysis of *rpoB’* gene sequences further supported this relationship, with GH36^T^ forming a robust clade (100% bootstrap) with *Haloarcula halophila* DFY41^T^, while also grouping with *Haloarcula pelagica* YJ-61-S^T^ and *Haloarcula litorea* GDY20^T^ in a distinct lineage (Figure S1b).

Collectively, both 16S rRNA and *rpoB’* gene analyses confirmed that GH36^T^ belongs to the genus *Haloarcula*. However, comprehensive genomic comparisons are required to definitively establish its taxonomic position at the species level.

### 3.2. ORGI and Phylogenomic Analyses

The complete genome assembly of strain GH36^T^ comprises a 2,975,322 bp chromosome and two plasmids (359,739 bp and 414,370 bp), totaling 3,749,431 bp with a GC content of 64.1%. Comparative genomic analysis revealed ANI, AAI, and dDDH values between GH36^T^ and other *Haloarcula* species ranging from 77.4–95.5%, 70.4–94.9%, and 25.2–67.1%, respectively (Tables S3–S5). Strain GH36^T^ showed the highest genomic relatedness to *Haloarcula halophila* DFY41^T^. Notably, all values fell below established species thresholds for prokaryotes (ANI 95–96%, AAI 95%, dDDH 70%) [24,38].

*Haloarcula amylolytica* JCM 13557^T^ and *Haloarcula hispanica* ATCC 33960^T^ exhibited an AAI value of 95.9%, and *Haloarcula argentinensis* DSM 12282^T^ and *Haloarcula sebkhae* JCM 19018^T^ showed 96.1% AAI, both exceeding the 95% species threshold. However, their ANI (94.4 and 94.5%) and dDDH (58.9 and 60.0%) values fell below established cutoff values (Tables S3–S5). This genomic divergence suggested that these strain pairs represented distinct species despite their high AAI. In contrast, three other type strains, *Haloarcula marismortui* ATCC 43049^T^, “*Haloarcula californiae*” ATCC 33799^T^, and “*Haloarcula sinaiiensis*” ATCC 33800^T^, demonstrated consistently high genomic relatedness, with ANI (97.8–98.3%), AAI (97.3–97.7%), and dDDH (85.0–86.4%) values all surpassing species delineation thresholds (Tables S3–S5). These results strongly indicated that these three strains should belong to a single species.

**Figure 1:**
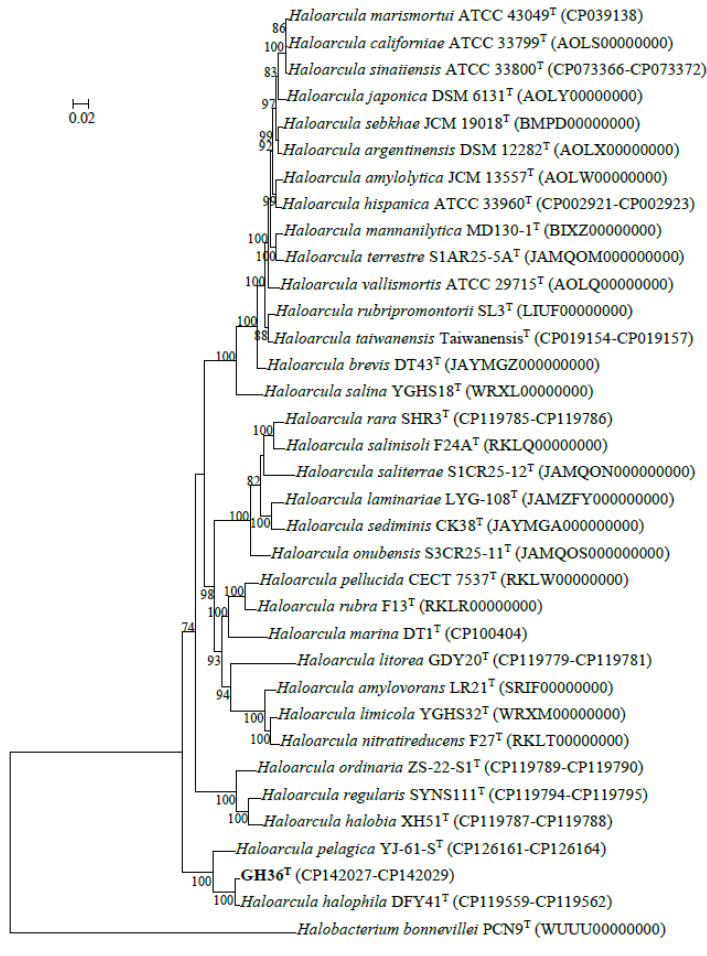
Maximum-likelihood phylogenetic tree constructed from concatenated gene sequences of 122 archaeal conserved marker proteins illustrating the evolutionary relationships among strain GH36^T^ and other species within the genus *Haloarcula*. Bootstrap support values (from 1000 replicates) are shown for branches with more than 70% support, and the scale bar indicates the expected number of substitutions per nucleotide position.

**Figure 2:**
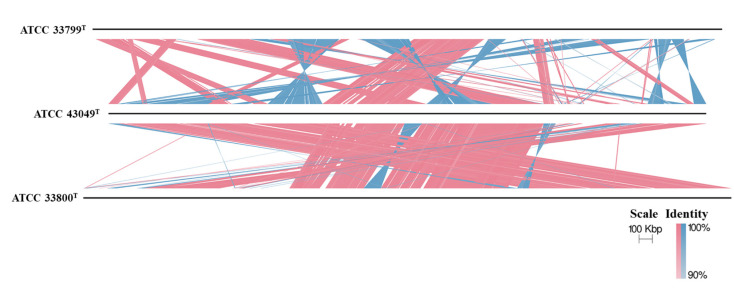
Synteny analysis between *Haloarcula marismortui* ATCC 43049^T^, “*Haloarcula californiae*” ATCC 33799^T^, and “*Haloarcula sinaiiensis*” ATCC 33800^T^. Only alignments of ≥500 bp and ≥90% identity are displayed. Vertical blocks between the genomes indicate regions of shared synteny, with red denoting matches in the same orientation and blue representing inverted matches.

Phylogenomic reconstruction using GTDB placed strain GH36^T^ in a well-supported cluster with *Haloarcula halophila* DFY41^T^ and *Haloarcula pelagica* YJ-61-S^T^ (Figure 1). These results collectively demonstrated that strain GH36^T^ represented a novel species within the genus *Haloarcula*. The strain pairs *Haloarcula amylolytica* JCM 13557^T^ and *Haloarcula hispanica* ATCC 33960^T^, as well as *Haloarcula argentinensis* DSM 12282^T^ and *Haloarcula sebkhae* JCM 19018^T^, each formed well-separated clades with long branch lengths, supporting their classification as distinct species (Figure 1). In contrast, *Haloarcula marismortui* ATCC 43049^T^, “*Haloarcula californiae*” ATCC 33799^T^, and “*Haloarcula sinaiiensis*” ATCC 33800^T^ clustered together with extremely short branch lengths, a topology consistent with their proposed reclassification as a single species based on genomic similarity thresholds (Figure 1). The synteny analysis between these three strains confirmed significant collinearity among the three strains, despite localized genomic rearrangements (Figure 2). Therefore, “*Haloarcula californiae*” ATCC 33799 and “*Haloarcula sinaiiensis*” ATCC 33800 are two reference strains of *Haloarcula marismortui*.

### 3.3. Genome Annotation and Comparative Genomic Analysis

RAST annotation predicted 4033 protein-coding genes and 47 tRNAs. In addition, subsystem categorization by RAST highlighted a pronounced functional focus on protein metabolism, along with the metabolism of amino acids and derivatives, as well as carbohydrates (Figure S2). Biotechnological screening of strain GH36^T^ identified a PHA biosynthesis gene cluster (encoding PhaR, PhaP, PhaE, PhaC, and PhaB), along with genes encoding putative PhaA homologs, indicating potential for PHA production (Figure S3).

**Figure 3:**
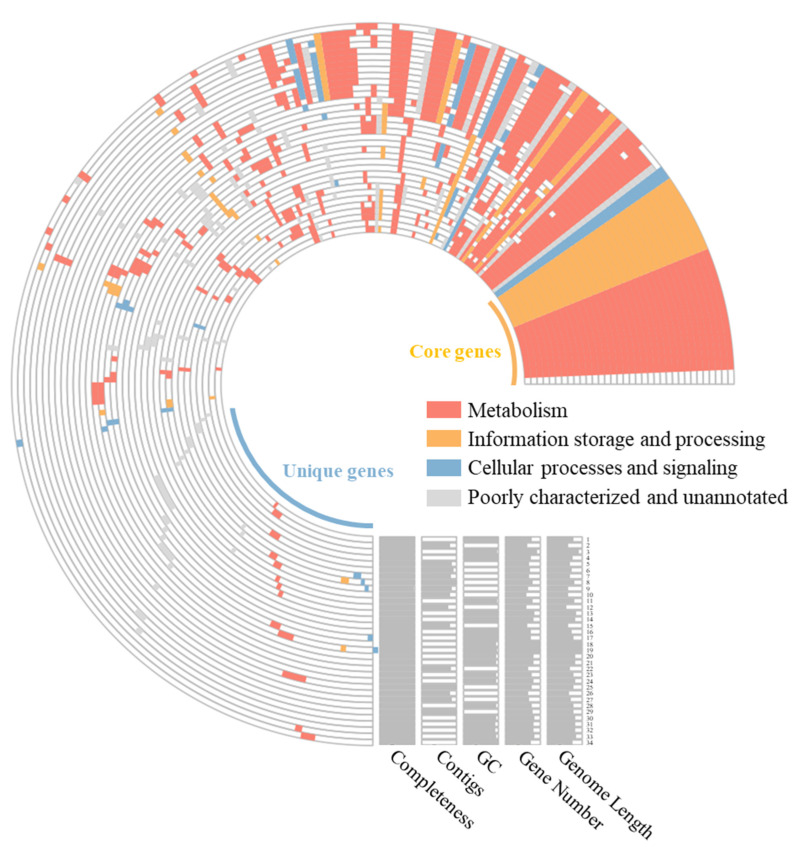
A comparative pan-genome analysis among strain GH36^T^ and the currently recognized species of *Haloarcula*. In the layers, colored areas denote the presence of specific gene clusters, while blank areas indicate their absence. 1, *Haloarcula pellucida*; 2, *Haloarcula ordinaria*; 3, *Haloarcula rubra*; 4, *Haloarcula marina*; 5, *Haloarcula litorea*; 6, *Haloarcula halophila*; 7, GH36^T^; 8, *Haloarcula pelagica*; 9, *Haloarcula regularis*; 10, *Haloarcula halobia*; 11, *Haloarcula salinisoli*; 12, *Haloarcula rara*; 13, *Haloarcula onubensis*; 14, *Haloarcula saliterrae*; 15, *Haloarcula sediminis*; 16, *Haloarcula laminariae*; 17, *Haloarcula limicola*; 18, *Haloarcula amylovorans*; 19, *Haloarcula nitratireducens*; 20, *Haloarcula terrestre*; 21, *Haloarcula mannanilytica*; 22, *Haloarcula brevis*; 23, *Haloarcula hispanica*; 24, *Haloarcula amylolytica*; 25, *Haloarcula californiae*; 26, *Haloarcula taiwanensis*; 27, *Haloarcula rubripromontorii*; 28, *Haloarcula vallismortis*; 29, *Haloarcula sinaiiensis*; 30, *Haloarcula marisnortui*; 31, *Haloarcula sebkhae*; 32, *Haloarcula argentinensis*; 33, *Haloarcula japonica*; 34, *Haloarcula salina*.

Pan-genome analysis of strain GH36^T^ along with other *Haloarcula* species revealed that as more genomes were included, the pan-genome expanded to 29,810 gene clusters while the core genome contracted to 1069 orthologous gene clusters before stabilizing (Figure S4). This trend indicated an open pan-genome architecture that facilitated the continuous acquisition of novel genetic material from the environment. Functional annotation using clusters of orthologous groups (COGs) categories showed that among the 29,810 pan-genome gene clusters, 3429 were related to metabolism, 2163 to cellular processes and signaling, and 1227 to information storage and processing, with an additional 19,191 gene clusters remaining poorly characterized or unannotated. Notably, strain GH36^T^ possessed 263 unique gene clusters that were not shared with other members of the genus (Figure 3).

**Figure 4:**
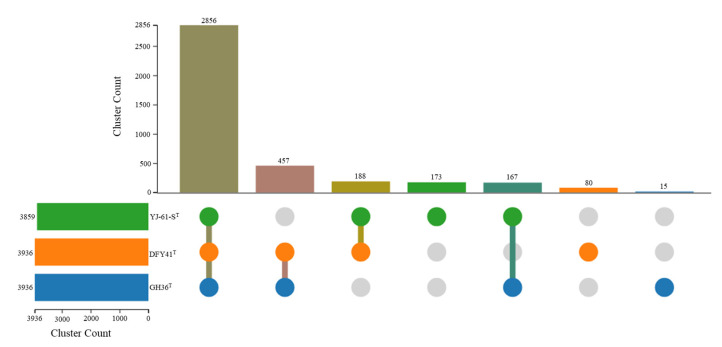
Comparative analysis of orthologous gene clusters for strains GH36^T^, *Haloarcula halophila* DFY41^T^, and *Haloarcula pelagica* YJ-61-S^T^.

OC analysis was conducted to examine the genomic relationships between strain GH36^T^ and its closest relatives, *Haloarcula halophila* DFY41^T^ and *Haloarcula pelagica* YJ-61-S^T^. The analysis identified 2856 core OCs shared among all three strains, 812 accessory OCs present in two strains, and 268 strain-specific clusters (15 in GH36^T^, 80 in DFY41^T^, and 173 in YJ-61-S^T^) (Figure 4). Gene Ontology (GO) enrichment analysis revealed distinct functional specializations among the unique gene clusters of each strain. *Haloarcula pelagica* YJ-61-S^T^ showed significant enrichment in plasma membrane components (GO: 0005886) and zinc ion binding (GO: 0008270); *Haloarcula halophila* DFY41^T^ exhibited enrichment in double-strand break repair via homologous recombination (GO: 0000724), starch binding (GO: 2001070), alkanesulfonate transport (GO: 0042918), misfolded or incompletely synthesized protein catabolic process (GO: 0006515), and GTP binding (GO: 0005525); while the unique clusters of strain GH36^T^ displayed enrichment in cytosine catabolic process (GO: 0006209). These findings suggested that while the three strains shared a substantial core genome, their unique gene clusters reflected distinct evolutionary adaptations to specific ecological niches or physiological requirements. The enrichment of cytosine catabolic processes in GH36^T^ may indicate specialized nucleic acid metabolism capabilities not present in its close relatives.

### 3.4. Phenotypic and Chemotaxonomic Characteristics

**Figure 5:**
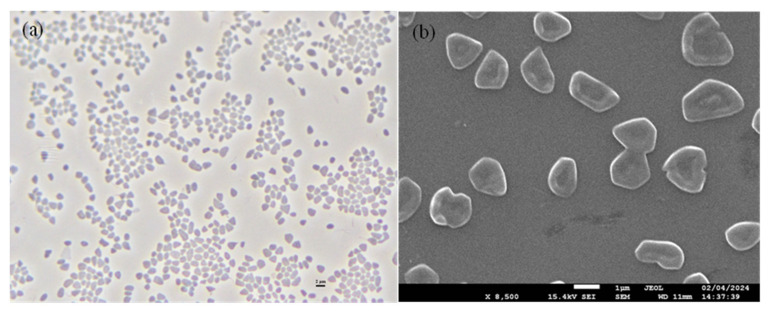
Phase-contrast micrograph (**a**) and scanning electron micrograph (**b**) of strain GH36^T^. Scale bar, 2 μm (**a**). Scale bar, 1 μm (**b**).

Strain GH36^T^ formed red-pigmented, moist, convex circular colonies with motile, pleomorphic cells (1.0–4.0 μm) that stained Gram-negative (Figure 5). The cells exhibited osmotic sensitivity, requiring a minimum of 0.86 M NaCl to prevent lysis in aqueous solutions. Optimal growth conditions were observed at 3.1 M NaCl (range: 2.6–5.1 M), 0.7 M MgCl_2_ (range: 0–1.0 M), 37 °C (range: 30–50 °C), and pH 7.5 (range: 6.5–9.0). While capable of anaerobic growth with nitrate or l-arginine as electron acceptors, the strain failed to use DMSO. Gas production was observed with nitrate as an electron acceptor. Biochemical characterization revealed negative reactions for hydrolysis of starch, casein, gelatin, and Tween 80, H_2_S production, indole formation, and oxidase activity, though catalase production was positive.

The strain demonstrated versatile carbon utilization, growing on various sugars (d-glucose, d-mannose, d-galactose, l-sorbose), sugar alcohols (d-sorbitol, d-mannitol, glycerol), organic acids (acetate, pyruvate, succinate, l-malate, fumarate, dl-lactate), and amino acid (l-glutamate). However, it could not metabolize d-fructose, pentoses (d-ribose, d-xylose), certain disaccharides (maltose, sucrose, lactose), citrate, or several amino acids (glycine, l-alanine, l-arginine, l-aspartate, l-lysine, l-ornithine). KEGG pathway annotation demonstrated that strain GH36^T^ possessed complete genetic machinery for central carbon metabolism, including key enzymes involved in glycolysis, the citrate cycle, and pyruvate oxidation. This metabolic machinery enabled the utilization of diverse organic substrates as carbon and energy sources. Notably, genomic analysis identified genes associated with glutamate metabolism, corroborated by phenotypic experiments confirming glutamate utilization as a growth substrate. Antibiotic susceptibility testing showed sensitivity to novobiocin, bacitracin, rifampin, nystatin, and nitrofurantoin, with resistance to other tested antibiotics (trimethoprim, erythromycin, penicillin G, ampicillin, chloramphenicol, neomycin, norfloxacin, ciprofloxacin, streptomycin, kanamycin, tetracycline, vancomycin, gentamicin, and nalidixic acid). Table 1 summarizes the key phenotypic differences between strain GH36^T^ and related *Haloarcula* species.

Chemotaxonomic analysis revealed a polar lipid profile characteristic of the genus *Haloarcula*, containing PG, PGP-Me, and PGS as major phospholipids (Figure S5). Two-dimensional TLC identified two glycolipids (GL1a and GL2a) chromatographically identical to S-DGD-1 and DGD-1, consistent with the chemotaxonomic markers of related *Haloarcula* species.

## 4. Taxonomic Conclusions

Comparative genomic analysis indicated that “*Haloarcula californiae*” ATCC 33799 and “*Haloarcula sinaiiensis*” ATCC 33800 served as reference strains for *Haloarcula marismortui*. Based on comprehensive polyphasic taxonomic characterization, strain GH36^T^ (=CGMCC 1.62631^T^ = MCCC 4K00122^T^) was proposed to be the type strain of a novel species, *Haloarcula montana* sp. nov.

### 4.1. Emended Description of Haloarcula marismortui (ex Volcani 1940) Oren et al. 1990

*Haloarcula marismortui* (ma.ris.mor’tu.i. L. gen. neut. n. *maris*, of the sea; L. adj. *mortuus -a -um*, dead; N.L. gen. neut. n. *marismortui*, of the Dead Sea).

The description is identical to that of *Haloarcula marismortui* as given previously [39]. The type strain is ATCC 43049^T^ (=CGMCC 1.1784^T^ = DSM 3752^T^), isolated from the Dead Sea. “*Haloarcula californiae*” ATCC 33799 (=BJGN-2 = JCM 8912) [11,40] isolated from a saltern at Guerrero Negro, Baja California, Mexico, and “*Haloarcula sinaiiensis*” ATCC 33800 (=BJSG-2) [11,40] isolated from the Red Sea sabkha (Sabkha Gavish), Baja California, Mexico, are two additional strains of *Haloarcula marismortui*.

### 4.2. Description of Haloarcula montana sp. nov.

*Haloarcula montana* (mon.ta’na. L. fem. adj. *montana*, of a mountain, found on mountains).

Cells are motile, pleomorphic (irregular granules, 1.0–4.0 µm) under optimal growth conditions and Gram-stain-negative. Colonies on agar plates are red, elevated, and round (colony size, diameter 0.5 mm). It can grow at 30–50 °C (optimum 37 °C), at 2.6–5.1 M NaCl (optimum 3.1 M), at 0–1.0 M MgCl_2_ (optimum 0.7 M), and at pH 6.5–9.0 (optimum pH 7.5). Cells lyse in distilled water, and the minimal NaCl concentration to prevent cell lysis is 0.86 M. Anaerobic growth occurs in the presence of nitrate and l-arginine, while anaerobic growth cannot be detected in the presence of DMSO. Nitrate reduction to nitrite was observed with the production of gas. Indole formation and H_2_S formation are negative. Catalase activity is positive, while oxidase activity is negative. Casein, gelatin, starch, or Tween 80 cannot be hydrolyzed. d-Glucose, d-mannose, d-galactose, l-sorbose, glycerol, d-mannitol, d-sorbitol, acetate, pyruvate, dl-lactate, succinate, l-malate, fumarate, and l-glutamate can be utilized as single carbon and energy sources for growth. No growth occurs on d-fructose, d-ribose, d-xylose, maltose, sucrose, lactose, citrate, glycine, l-alanine, l-arginine, l-aspartate, l-lysine, or l_-_ornithine. Acid is produced from d-glucose, d-mannose, d-galactose, l-sorbose, glycerol, d-mannitol, and d-sorbitol. The major polar lipids are PG, PGP-Me, PGS, S-DGD-1, and DGD-1.

The type strain GH36^T^ (=CGMCC 1.62631^T^ = MCCC 4K00122^T^) was isolated from Gahai Salt Lake, Qinghai, China. The DNA G+C content is 64.1% (genome). The GenBank/EMBL/DDBJ accession numbers for the 16S rRNA gene, *rpoB′* gene, and whole genome sequences of strain GH36^T^ are MZ463760 (*rrnA*), OQ518442 (*rrnB*), PP070407, and CP142027–CP142029, respectively.

## Figures and Tables

**Table 1:** Differential characteristics of strain GH36^T^ and closely related species within the genus *Haloarcula*. Taxa: 1, GH36^T^; 2, *Haloarcula sediminis* CK38^T^; 3, *Haloarcula brevis* DT43^T^; 4, *Haloarcula regularis* SYNS111^T^; 5, *Haloarcula amylolytica* BD-3^T^; 6, *Haloarcula halobia* XH51^T^; 7, *Haloarcula halophila* DFY41^T^; 8, *Haloarcula laminariae* LYG-108^T^; 9, *Haloarcula ordinaria* ZS-22-S1^T^; 10, *Haloarcula pelagica* YJ-61-S^T^. Symbols: +, positive; −, negative.

Characteristic	1	2	3	4	5	6	7	8	9	10
Optimum NaCl (M)	3.1	2.6	4.8	3.4	3.1	3.4	4.8	2.6	2.6	3.4
Temperature optimum (℃)	37	42	40	37	41	35	37	40	37	35
Optimum pH	7.5	7.0	7.0	7.0	7.5	7.0	7.0	7.0	7.0	7.0
Anaerobic growth with nitrate	+	+	+	+	+	−	−	−	−	−
Anaerobic growth with arginine	+	+	−	−	−	−	−	−	−	−
Anaerobic growth with DMSO	−	+	−	+	−	−	−	−	−	+
Utilization of:										
d-Mannose	+	+	+	+	+	−	+	+	+	−
d-Galactose	+	+	+	+	+	+	+	+	−	+
l-Sorbose	+	−	−	−	−	−	−	−	−	−
Maltose	−	+	+	−	+	−	−	−	−	−
Sucrose	−	+	+	+	+	+	+	+	+	+
Lactose	−	+	+	−	−	−	−	−	−	−
Indole formation	−	−	−	−	+	−	−	−	−	−
Starch hydrolysis	−	+	−	−	+	−	−	−	−	+
Gelatin hydrolysis	−	−	−	−	−	−	−	−	−	−
Tween 80 hydrolysis	−	−	−	−	−	−	−	−	−	+
H_2_S formation	−	+	−	−	+	−	−	+	+	+

## Data Availability

The GenBank accession numbers for the 16S rRNA gene, *rpoB′* gene, and whole genome sequences of strain GH36^T^ are MZ463760 (*rrnA*), OQ518442 (*rrnB*), PP070407, and CP142027–CP142029, respectively.

## Supplementary Materials

The following supporting information can be downloaded at https://www.mdpi.com/article/10.3390/biology14060615/s1, Table S1. 16S rRNA gene sequence similarities among strain GH36^T^ and current species of the genus *Haloarcula*. Table S2. *rpoB’* gene sequence similarities among strain GH36^T^ and the current species of the genus *Haloarcula*. Table S3. Pairwise ANI values among strain GH36^T^ and the current species of the genus *Haloarcula*. Table S4. Pairwise AAI values among strain GH36^T^ and the current species of the genus *Haloarcula*. Table S5. Pairwise dDDH values among strain GH36^T^ and the current species of the genus *Haloarcula*. Figure S1. Maximum-likelihood phylogenetic trees reconstructed using 16S rRNA (**a**) and *rpoB’* (**b**) gene sequences to elucidate the evolutionary relationships among strain GH36^T^ and the currently recognized species of *Haloarcula*. Figure S2. Functional annotation of the genome of strain GH36^T^ by using the online Rapid Annotation Subsystems Technology (RAST) server. Figure S3. The gene cluster for PHA biosynthesis in strain GH36^T^. Figure S4. Gene cluster accumulation curve of the pan-genome (blue) and the core-genome (orange) of species of the genus *Haloarcula*. Figure S5. Analysis of the polar lipid composition of strain GH36^T^ using thin-layer chromatography (TLC).
