# Supplementary material for: Genome-Based Reclassification of Two Haloarcula Species and Characterization of Haloarcula montana sp. nov
Source: Biology (Basel). 2025 May 27;14(6):615. doi: 10.3390/biology14060615 (PMC12189638; doi:10.3390/biology14060615)
Supplement: Supplementary file 1 [file biology-14-00615-s001.zip › biology-3603415-supplementary.pdf]

Table S1. 16S rRNA gene sequence similarities among strain GH36^{T} and current species of the genus *Haloarcula*. Values exceeding the species-level threshold of 98.65% are highlighted in yellow.

No.	16S rRNA gene sequence similarity (%)		1		2			3		4		5		6		7		8		9			10	
			rrnA	rrnB	rrnA	rrnB	rrnC	rrnA	rrnB	rrnA	rrnB	rrnA	rrnB	rrnA	rrnB	rrnA	rrnB	rrnA	rrnB	rrn1	rrn2	rrn3	rrnA	rrnB
1	Strain GH36^{T}	rrnA	*	93.2	93.9	91.0	90.7	93.8	91.1	93.8	90.7	94.1	91.2	90.7	94.2	99.9	93.1	92.7	90.4	91.4	91.1	94.2	94.4	91.2
		rrnB	93.2	*	93.5	94.6	94.0	94.4	96.3	93.3	94.1	93.8	94.8	94.1	93.6	93.1	99.9	93.5	96.6	94.4	94.2	93.6	93.4	94.5
2	*Haloarcula amylolytica* JCM 13557^{T}	rrnA	93.9	93.5	*	95.1	94.4	95.7	93.0	98.6	94.6	98.4	94.8	94.2	99.0	93.9	93.4	90.6	92.9	94.8	94.6	99.2	98.6	95.0
		rrnB	91.0	94.6	95.1	*	98.6	92.6	95.2	95.1	98.3	95.2	98.2	98.3	94.8	91.0	94.5	90.5	94.5	98.9	98.6	95.0	95.2	99.3
		rrnC	90.7	94.0	94.4	98.6	*	92.3	95.0	94.5	99.1	95.0	98.4	98.9	94.0	90.7	94.0	90.6	94.6	98.8	99.1	94.3	94.6	99.2
3	*Haloarcula amylovorans* LR21^{T}	rrnA	93.8	94.4	95.7	92.6	92.3	*	95.7	95.4	92.3	96.1	93.0	92.4	95.8	93.8	94.4	95.7	93.5	92.9	92.6	95.9	95.6	92.6
		rrnB	91.1	96.3	93.0	95.2	95.0	95.7	*	92.7	95.1	93.3	95.9	95.0	92.9	91.0	96.2	92.7	96.0	95.5	95.2	92.9	92.7	95.2
4	*Haloarcula argentinensis* DSM 12282^{T}	rrnA	93.8	93.3	98.6	95.1	94.5	95.4	92.7	*	95.2	98.5	95.0	94.4	99.0	93.8	93.2	93.7	92.9	95.2	95.0	98.9	98.8	95.0
		rrnB	90.7	94.1	94.6	98.3	99.1	92.3	95.1	95.2	*	95.3	98.5	99.0	94.7	90.7	94.0	91.0	94.9	98.9	99.2	94.6	94.9	98.8
5	*Haloarcula brevis* DT43^{T}	rrnA	94.1	93.8	98.4	95.2	95.0	96.1	93.3	98.5	95.3	*	96.3	95.0	98.7	94.1	93.8	94.2	93.3	95.1	95.4	98.7	98.6	95.0
		rrnB	91.2	94.8	94.8	98.2	98.4	93.0	95.9	95.0	98.5	96.3	*	98.5	94.9	91.2	94.7	91.3	95.2	98.5	98.8	95.0	95.0	98.1
6	*Haloarcula californiae* ATCC 33799^{T}	rrnA	90.7	94.1	94.2	98.3	98.9	92.4	95.0	94.4	99.0	95.0	98.5	*	94.6	90.7	94.1	90.6	94.6	98.4	96.7	94.1	94.6	98.6
		rrnB	94.2	93.6	99.0	94.8	94.0	95.8	92.9	99.0	94.7	98.7	94.9	94.6	*	94.2	93.5	94.0	92.9	96.0	96.5	99.2	98.7	94.6
7	*Haloarcula halophila* DFY41^{T}	rrnA	99.9	93.1	93.9	91.0	90.7	93.8	91.0	93.8	90.7	94.1	91.2	90.7	94.2	*	93.1	92.2	90.3	91.4	91.1	94.2	94.4	91.2
		rrnB	93.1	99.9	93.4	94.5	94.0	94.4	96.2	93.2	94.0	93.8	94.7	94.1	93.5	93.1	*	96.5	92.7	94.4	94.1	93.5	94.2	94.4
8	*Haloarcula halobia* XH51^{T}	rrnA	92.7	93.5	90.6	90.5	90.6	95.7	92.7	93.7	91.0	94.2	91.3	90.6	94.0	92.2	96.5	*	94.6	91.0	91.0	94.0	93.3	90.5
		rrnB	90.4	96.6	92.9	94.5	94.6	93.5	96.0	92.9	94.9	93.3	95.2	94.6	92.9	90.3	92.7	94.6	*	95.0	95.0	92.9	92.5	94.5
9	*Haloarcula hispanica* ATCC 33960^{T}	rrn1	91.4	94.4	94.8	98.9	98.8	92.9	95.5	95.2	98.9	95.1	98.5	98.4	96.0	91.4	94.4	91.0	95.0	*	99.7	95.0	95.5	99.1
		rrn2	91.1	94.2	94.6	98.6	99.1	92.6	95.2	95.0	99.2	95.4	98.8	96.7	96.5	91.1	94.1	91.0	95.0	99.7	*	94.8	95.2	98.8
		rrn3	94.2	93.6	99.2	95.0	94.3	95.9	92.9	98.9	94.6	98.7	95.0	94.1	99.2	94.2	93.5	94.0	92.9	95.0	94.8	*	98.9	95.0
10	*Haloarcula japonica* DSM 6131^{T}	rrnA	94.4	93.4	98.6	95.2	94.6	95.6	92.7	98.8	94.9	98.6	95.0	94.6	98.7	94.4	94.2	93.3	92.5	95.5	95.2	98.9	*	95.3
		rrnB	91.2	94.5	95.0	99.3	99.2	92.6	95.2	95.0	98.8	95.0	98.1	98.6	94.6	91.2	94.4	90.5	94.5	99.1	98.8	95.0	95.3	*
		rrnC	91.1	94.2	94.6	98.4	99.1	92.7	95.4	94.6	99.1	95.1	98.5	99.2	94.3	91.1	94.2	90.8	94.8	99.2	99.5	94.5	94.9	98.8
11	*Haloarcula laminariae* LYG-108^{T}	rrnA	93.6	92.1	94.9	92.5	91.9	94.9	92.2	94.9	92.3	95.4	92.7	92.1	95.2	93.5	92.0	94.7	90.8	92.5	92.3	95.2	94.8	92.5
		rrnB	90.1	93.7	92.7	95.2	95.5	93.0	95.0	93.1	95.9	94.0	96.3	95.9	92.9	90.0	93.6	90.5	93.7	95.7	95.9	92.9	93.1	95.2
12	*Haloarcula limicola* YGHS32^{T}	rrnA	93.5	94.5	96.1	92.7	92.1	99.0	95.4	95.9	92.4	96.4	92.9	91.8	96.1	93.4	94.4	96.0	93.5	92.6	92.3	96.3	95.7	92.7
		rrnB	90.8	95.8	92.9	95.5	94.9	95.1	98.8	92.9	95.0	93.3	95.5	94.7	92.8	90.7	95.7	92.5	95.6	95.4	95.1	92.9	92.7	95.5
13	*Haloarcula litorea* GDY20^{T}	rrnA	95.1	91.8	94.4	91.2	91.1	94.4	91.7	94.1	91.2	94.3	91.4	90.4	94.3	95.1	91.7	93.3	91.0	91.4	91.2	94.5	94.2	91.5
		rrnB	91.0	94.1	94.9	92.9	94.8	92.7	95.1	94.6	93.0	93.3	95.3	94.5	92.9	91.0	94.0	92.8	94.3	94.8	95.2	95.0	92.9	95.0
14	*Haloarcula mannanilytica* MD-130^{T}	rrn1	94.1	93.5	99.0	95.1	94.5	95.8	93.0	99.0	94.6	99.1	95.4	94.6	99.0	94.1	93.4	93.8	92.8	95.0	94.7	99.4	99.0	95.0
		rrn2	93.8	93.5	98.9	95.4	94.6	95.8	93.0	98.9	94.9	99.1	95.4	94.8	98.7	93.8	93.4	93.7	92.8	95.2	95.0	99.0	98.7	95.3
		rrn3	90.9	94.4	95.0	98.2	98.9	92.5	95.4	98.8	99.0	95.8	99.1	99.1	94.6	90.9	94.3	90.8	94.8	98.7	99.0	95.0	95.2	98.7
15	*Haloarcula marina* DT1^{T}	rrnA	93.1	92.9	94.6	91.1	90.8	93.2	92.7	94.5	90.8	94.3	91.1	91.0	94.8	93.1	92.8	96.1	92.2	91.3	91.0	94.8	94.4	91.2
		rrnB	89.7	95.4	92.1	95.0	95.0	92.7	95.9	92.3	94.9	92.5	95.4	94.8	92.0	89.7	95.3	91.4	95.2	95.2	94.9	92.0	92.0	95.2
16	*Haloarcula marismortui* ATCC 43049^{T}	rrnA	94.2	93.8	99.3	95.0	94.4	96.0	93.1	94.7	94.7	98.6	95.0	94.6	99.6	94.2	93.7	94.3	93.1	95.0	94.7	99.4	98.6	94.9
		rrnB	91.0	94.3	94.6	98.4	99.1	92.5	95.2	94.6	99.3	95.2	98.6	99.5	94.8	91.0	94.2	90.8	94.7	99.0	99.3	94.4	94.9	98.9
		rrnC	90.8	94.1	94.2	98.6	99.2	92.2	94.8	94.6	99.2	94.9	98.2	99.4	94.1	90.8	94.0	90.5	94.7	98.9	99.2	94.2	94.6	98.9
17	*Haloarcula nitratireducens* F27^{T}	rrnA	93.6	94.2	96.1	92.7	92.1	98.7	95.1	96.1	92.5	96.5	92.9	92.3	96.6	93.5	95.3	95.8	93.3	92.9	92.6	96.4	95.7	92.5
		rrnB	89.5	95.1	91.7	93.8	93.7	93.7	96.5	92.0	93.8	92.2	93.9	93.6	91.8	89.4	97.2	92.0	95.5	94.0	93.8	91.7	91.5	94.1
18	*Haloarcula onubensis* S3CR25-11^{T}	rrnA	90.8	94.4	93.2	95.4	95.4	93.0	95.1	93.2	95.8	94.1	96.0	95.9	92.6	90.7	94.3	91.0	94.0	95.5	95.8	93.5	93.2	95.6
		rrnB	93.4	91.6	94.5	92.1	92.1	94.0	91.3	94.5	92.5	95.4	92.6	92.5	94.9	93.4	91.5	95.0	91.1	92.3	92.6	94.8	94.2	91.9
19	*Haloarcula ordinaria* ZS-22-S1^{T}	rrnA	94.3	92.3	95.3	92.0	91.4	95.3	92.3	95.3	91.8	95.7	92.3	91.3	95.7	94.3	92.3	97.1	92.0	92.2	91.9	95.5	95.0	91.8
		rrnB	91.8	96.8	94.2	95.9	95.2	94.1	96.8	94.0	95.4	94.5	96.1	95.1	94.0	92.9	96.7	92.7	95.4	95.9	95.6	94.3	94.0	95.8
20	*Haloarcula pelagica* YJ-61-S^{T}	rrnA	99.3	92.7	94.3	91.4	91.0	94.0	91.4	94.2	91.1	94.4	91.6	91.0	94.6	99.3	92.7	93.1	90.6	91.6	91.4	94.4	94.6	91.5
		rrnB	92.6	98.4	93.5	94.5	94.2	94.4	95.9	93.1	94.2	93.8	94.7	94.1	93.5	92.5	98.3	92.9	96.0	94.3	94.0	93.5	93.1	94.6
21	*Haloarcula pellucida* CECT 7537^{T}	rrnA	93.2	92.9	95.9	93.2	92.5	95.9	92.7	96.3	92.7	96.0	92.9	92.6	96.4	93.2	94.2	94.4	92.2	93.1	92.8	96.5	95.8	93.0
		rrnB	91.3	95.2	93.6	96.5	95.7	92.6	95.1	93.7	95.7	93.7	95.7	95.7	93.4	91.2	95.1	90.7	94.1	96.1	95.8	93.7	93.4	96.3
22	*Haloarcula rara* SHR3^{T}	rrnA	93.3	91.8	94.8	92.5	91.6	94.1	91.6	94.5	91.9	95.0	92.4	92.1	95.0	93.3	91.7	94.2	90.4	92.3	92.1	94.9	94.5	92.3
		rrnB	91.0	93.8	93.0	95.4	95.5	92.3	91.7	93.1	95.9	94.0	95.9	96.2	93.2	91.0	93.8	90.1	93.0	95.6	95.9	93.1	93.3	95.7
23	*Haloarcula regularis* SYNS111^{T}	rrnA	92.9	93.6	94.1	90.8	90.8	95.8	92.8	93.8	91.0	94.6	91.6	90.6	94.1	92.9	93.5	99.6	94.4	91.0	91.0	94.3	93.7	90.8
		rrnB	90.5	96.9	92.9	94.6	94.6	93.4	96.3	92.7	94.8	93.3	95.3	94.5	92.6	90.5	96.8	94.2	99.5	94.8	94.8	92.9	92.6	94.6
24	*Haloarcula rubra* F13^{T}	rrnA	94.1	93.3	95.0	91.9	91.3	95.7	92.9	95.0	91.7	94.9	91.9	90.9	95.1	94.2	93.4	96.0	92.3	91.7	91.4	95.3	94.8	91.8
		rrnB	90.2	95.9	92.2	94.9	94.2	93.2	96.4	92.2	94.4	92.4	94.9	94.2	92.1	90.1	95.8	91.9	95.8	94.7	94.4	92.3	92.0	94.8
25	*Haloarcula rubripromontorii* SL3^{T}	rrnA	94.0	93.2	98.9	94.8	94.7	95.9	93.1	98.6	95.1	98.4	95.0	94.9	98.8	94.0	93.1	93.9	93.0	96.4	96.8	99.1	98.7	95.1
		rrnB	91.0	94.5	95.3	98.8	98.0	92.3	95.2	95.6	98.2	95.5	98.5	98.3	95.2	91.0	94.4	90.7	94.6	98.4	96.5	95.6	95.4	98.6
26	*Haloarcula salina* YGHS18^{T}	rrnA	93.5	94.0	96.0	94.1	93.6	96.0	93.9	96.4	93.8	96.7	94.2	93.0	96.1	93.5	93.9	94.0	93.5	94.0	93.7	96.3	96.4	94.2
		rrnB	91.2	95.1	93.1	96.8	96.3	93.1	95.8	93.7	96.3	94.0	96.9	95.8	93.2	91.2	95.0	91.2	94.8	96.7	96.4	93.5	93.6	96.9
27	*Haloarcula salinisoli* F24A^{T}	rrnA	93.2	91.3	94.3	91.9	91.0	94.0	91.1	94.2	91.4	94.5	91.8	91.7	94.7	93.2	91.2	93.9	89.9	91.7	91.4	94.5	94.1	91.7
		rrnB	90.9	93.9	93.4	95.7	95.7	92.9	95.0	93.4	95.9	93.9	95.8	95.9	93.1	91.3	94.3	91.0	93.9	96.1	96.3	93.7	93.6	95.8
28	*Halomicroarcula saliterrae* S1CR25-12^{T}	rrnA	93.9	92.1	95.2	92.4	91.7	95.1	92.0	95.2	92.1	95.6	92.4	91.8	95.6	93.9	92.1	95.9	91.4	92.6	92.3	95.6	94.9	92.1
		rrnB	90.6	94.3	92.7	95.4	96.2	92.7	94.8	92.7	96.3	94.0	96.5	96.2	92.8	90.5	94.3	90.7	93.7	95.9	96.2	92.9	93.1	95.9
29	*Haloarcula sebkhae* JCM 19018^{T}	rrnA	93.7	92.9	98.9	94.8	94.1	95.0	92.9	99.2	94.8	98.1	94.3	93.8	99.1	94.1	93.3	94.1	93.1	95.0	94.7	99.4	98.7	94.6
		rrnB	90.2	93.6	93.8	98.0	98.6	91.8	94.4	98.0	98.6	94.5	97.9	98.7	94.1	94.1	93.6	90.1	94.2	98.3	98.6	93.8	94.0	98.3
30	*Haloarcula sediminis* CK38^{T}	rrnA	93.8	92.0	94.8	92.1	92.3	94.9	92.2	94.5	92.4	95.1	92.7	92.5	94.9	93.8	91.9	94.6	90.7	92.7	92.4	95.0	94.8	92.5
		rrnB	90.8	94.1	93.4	95.7	95.7	92.9	95.1	93.5	96.0	93.8	96.1	96.3	93.6	90.7	94.0	90.4	93.3	96.3	96.0	93.8	93.8	96.1
31	*Haloarcula sinaiiensis* ATCC 33800^{T}	rrnA	90.5	94.0	93.9	97.8	98.4	91.9	94.6	94.0	98.7	94.6	97.9	99.0	93.9	90.5	93.9	90.1	94.3	97.9	96.3	93.7	94.3	98.2
		rrnB	91.4	93.3	95.7	96.9	96.9	93.7	93.8	95.7	97.1	96.7	97.3	97.1	95.9	91.4	93.3	92.0	93.7	97.6	95.0	95.6	95.9	96.7
32	*Haloarcula taiwanensis* Taiwanensis^{T}	rrn1	93.8	93.2	98.8	95.5	94.7	95.5	92.7	99.2	94.8	98.7	95.0	94.5	98.8	93.8	93.1	93.6	92.6	95.0	94.8	99.2	98.9	95.4
		rrn2	90.6	94.1	94.5	98.6	99.2	92.2	95.1	95.0	99.2	95.5	98.8	99.0	94.3	90.6	94.0	90.6	94.6	98.8	99.1	94.7	94.9	99.1
33	*Haloarcula terrestris* S1AR25-5A^{T}	rrnA	94.3	93.6	99.3	95.0	94.3	95.9	93.1	99.0	94.7	98.9	95.2	94.2	99.5	94.3	93.6	94.0	92.9	94.9	94.6	99.7	99.1	95.0
		rrnB	89.7	95.1	93.3	99.3	99.1	92.1	96.1	93.6	99.4	94.0	98.9	99.2	93.0	89.7	95.0	89.5	95.6	99.6	99.6	93.3	94.0	99.5
34	*Haloarcula vallismortis* ATCC 29715^{T}	rrnA	94.0	93.6	98.6	94.8	93.9	95.5	92.9	94.8	94.5	98.2	94.8	94.1	98.6	94.0	93.6	93.6	93.1	94.8	94.5	98.9	98.4	94.6
		rrnB	91.0	94.2	94.0	98.0	98.7	92.4	95.0	98.5	99.1	95.2	98.5	99.3	94.3	91.0	94.1	90.8	94.8	98.8	99.1	94.3	94.8	98.4

No.	16S rRNA gene sequence similarity (%)		10	11		12		13		14			15		16			17		18		19		20		21		22
			rrnC	rrnA	rrnB	rrnA	rrnB	rrnA	rrnB	rrn1	rrn2	rrn3	rrnA	rrnB	rrnA	rrnB	rrnC	rrnA	rrnB	rrnA	rrnB	rrnA	rrnB	rrnA	rrnB	rrnA	rrnB	rrnA
1	Strain GH36^{T}	rrnA	91.1	93.6	90.1	93.5	90.8	95.1	91.0	94.1	93.8	90.9	93.1	89.7	94.2	91.0	90.8	93.6	89.5	90.8	93.4	94.3	91.8	99.3	92.6	93.2	91.3	93.3
		rrnB	94.2	92.1	93.7	94.5	95.8	91.8	94.1	93.5	93.5	94.4	92.9	95.4	93.8	94.3	94.1	94.2	95.1	94.4	91.6	92.3	96.8	92.7	98.4	92.9	95.2	91.8
2	*Haloarcula amylolytica* JCM 13557^{T}	rrnA	94.6	94.9	92.7	96.1	92.9	94.4	94.9	99.0	98.9	95.0	94.6	92.1	99.3	94.6	94.2	96.1	91.7	93.2	94.5	95.3	94.2	94.3	93.5	95.9	93.6	94.8
		rrnB	98.4	92.5	95.2	92.7	95.5	91.2	92.9	95.1	95.4	98.2	91.1	95.0	95.0	98.4	98.6	92.7	93.8	95.4	92.1	92.0	95.9	91.4	94.5	93.2	96.5	92.5
		rrnC	99.1	91.9	95.5	92.1	94.9	91.1	94.8	94.5	94.6	98.9	90.8	95.0	94.4	99.1	99.2	92.1	93.7	95.4	92.1	91.4	95.2	91.0	94.2	92.5	95.7	91.6
3	*Haloarcula amylovorans* LR21^{T}	rrnA	92.7	94.9	93.0	99.0	95.1	94.4	92.7	95.8	95.8	92.5	93.2	92.7	96.0	92.5	92.2	98.7	93.7	93.0	94.0	95.3	94.1	94.0	94.4	95.9	92.6	94.1
		rrnB	95.4	92.2	95.0	95.4	98.8	91.7	95.1	93.0	93.0	95.4	92.7	95.9	93.1	95.2	94.8	95.1	96.5	95.1	91.3	92.3	96.8	91.4	95.9	92.7	95.1	91.6
4	*Haloarcula argentinensis* DSM 12282^{T}	rrnA	94.6	94.9	93.1	95.9	92.9	94.1	94.6	99.0	98.9	98.8	94.5	92.3	94.7	94.6	94.6	96.1	92.0	93.2	94.5	95.3	94.0	94.2	93.1	96.3	93.7	94.5
		rrnB	99.1	92.3	95.9	92.4	95.0	91.2	93.0	94.6	94.9	99.0	90.8	94.9	94.7	99.3	99.2	92.5	93.8	95.8	92.5	91.8	95.4	91.1	94.2	92.7	95.7	91.9
5	*Haloarcula brevis* DT43^{T}	rrnA	95.1	95.4	94.0	96.4	93.3	94.3	93.3	99.1	99.1	95.8	94.3	92.5	98.6	95.2	94.9	96.5	92.2	94.1	95.4	95.7	94.5	94.4	93.8	96.0	93.7	95.0
		rrnB	98.5	92.7	96.3	92.9	95.5	91.4	95.3	95.4	95.4	99.1	91.1	95.4	95.0	98.6	98.2	92.9	93.9	96.0	92.6	92.3	96.1	91.6	94.7	92.9	95.7	92.4
6	*Haloarcula californiae* ATCC 33799^{T}	rrnA	99.2	92.1	95.9	91.8	94.7	90.4	94.5	94.6	94.8	99.1	91.0	94.8	94.6	99.5	99.4	92.3	93.6	95.9	92.5	91.3	95.1	91.0	94.1	92.6	95.7	92.1
		rrnB	94.3	95.2	92.9	96.1	92.8	94.3	92.9	99.0	98.7	94.6	94.8	92.0	99.6	94.8	94.1	96.6	91.8	92.6	94.9	95.7	94.0	94.6	93.5	96.4	93.4	95.0
7	*Haloarcula halophila* DFY41^{T}	rrnA	91.1	93.5	90.0	93.4	90.7	95.1	91.0	94.1	93.8	90.9	93.1	89.7	94.2	91.0	90.8	93.5	89.4	90.7	93.4	94.3	92.9	99.3	92.5	93.2	91.2	93.3
		rrnB	94.2	92.0	93.6	94.4	95.7	91.7	94.0	93.4	93.4	94.3	92.8	95.3	93.7	94.2	94.0	95.3	97.2	94.3	91.5	92.3	96.7	92.7	98.3	94.2	95.1	91.7
8	*Haloarcula halobia* XH51^{T}	rrnA	90.8	94.7	90.5	96.0	92.5	93.3	92.8	93.8	93.7	90.8	96.1	91.4	94.3	90.8	90.5	95.8	92.0	91.0	95.0	97.1	92.7	93.1	92.9	94.4	90.7	94.2
		rrnB	94.8	90.8	93.7	93.5	95.6	91.0	94.3	92.8	92.8	94.8	92.2	95.2	93.1	94.7	94.7	93.3	95.5	94.0	91.1	92.0	95.4	90.6	96.0	92.2	94.1	90.4
9	*Haloarcula hispanica* ATCC 33960^{T}	rrn1	99.2	92.5	95.7	92.6	95.4	91.4	94.8	95.0	95.2	98.7	91.3	95.2	95.0	99.0	98.9	92.9	94.0	95.5	92.3	92.2	95.9	91.6	94.3	93.1	96.1	92.3
		rrn2	99.5	92.3	95.9	92.3	95.1	91.2	95.2	94.7	95.0	99.0	91.0	94.9	94.7	99.3	99.2	92.6	93.8	95.8	92.6	91.9	95.6	91.4	94.0	92.8	95.8	92.1
		rrn3	94.5	95.2	92.9	96.3	92.9	94.5	95.0	99.4	99.0	95.0	94.8	92.0	99.4	94.4	94.2	96.4	91.7	93.5	94.8	95.5	94.3	94.4	93.5	96.5	93.7	94.9
10	*Haloarcula japonica* DSM 6131^{T}	rrnA	94.9	94.8	93.1	95.7	92.7	94.2	92.9	99.0	98.7	95.2	94.4	92.0	98.6	94.9	94.6	95.7	91.5	93.2	94.2	95.0	94.0	94.6	93.1	95.8	93.4	94.5
		rrnB	98.8	92.5	95.2	92.7	95.5	91.5	95.0	95.0	95.3	98.7	91.2	95.2	94.9	98.9	98.9	92.5	94.1	95.6	91.9	91.8	95.8	91.5	94.6	93.0	96.3	92.3
		rrnC	*	92.1	95.8	92.3	95.0	90.9	94.6	94.6	94.8	99.0	91.2	95.0	94.7	99.4	99.1	92.5	94.0	95.7	92.5	91.8	95.5	91.4	94.3	92.7	95.7	92.1
11	*Haloarcula laminariae* LYG-108^{T}	rrnA	92.1	*	94.4	95.3	92.5	94.3	92.6	95.5	95.5	92.5	95.2	91.0	95.1	92.1	91.9	94.9	90.6	94.3	98.0	96.4	92.4	94.0	92.7	95.0	92.7	98.8
		rrnB	95.8	94.4	*	92.9	95.0	90.7	94.6	93.5	93.5	96.1	91.1	94.1	92.8	95.9	95.7	92.9	93.4	97.5	93.2	91.3	94.4	90.1	93.8	93.2	95.9	93.3
12	*Haloarcula limicola* YGHS32^{T}	rrnA	92.3	95.3	92.9	*	95.9	94.6	93.2	96.1	96.1	92.4	95.9	92.6	96.3	92.1	91.8	95.3	97.2	93.4	94.6	95.9	94.6	93.8	94.7	92.9	95.5	94.4
		rrnB	95.0	92.5	95.0	95.9	*	91.6	95.0	92.9	92.9	94.9	92.5	95.7	93.1	94.9	94.7	95.3	97.2	95.4	91.7	92.5	96.9	91.0	95.9	92.9	95.5	91.8
13	*Haloarcula litorea* GDY20^{T}	rrnA	90.9	94.3	90.7	94.6	91.6	*	93.9	94.4	94.1	91.1	95.2	91.1	94.6	90.8	90.8	94.4	90.5	91.2	94.3	95.2	92.9	95.4	91.2	94.5	91.4	93.8
		rrnB	94.6	92.6	94.6	93.2	95.0	93.9	*	93.1	93.1	95.0	92.8	93.3	93.3	94.6	94.5	93.8	93.4	95.0	92.7	92.2	93.7	91.2	93.3	95.1	92.9	92.2
14	*Haloarcula mannanilytica* MD-130^{T}	rrn1	94.6	95.5	93.5	96.1	92.9	94.4	93.1	*	99.5	95.5	95.1	92.3	99.2	94.6	94.5	96.3	92.0	93.6	95.1	95.5	94.2	94.4	93.5	96.5	93.9	95.1
		rrn2	94.8	95.5	93.5	96.1	92.9	94.1	93.1	99.5	*	95.8	94.6	95.0	98.8	94.8	94.8	96.2	92.0	93.7	95.0	95.5	94.2	94.2	93.5	96.3	94.0	95.2
		rrn3	99.0	92.5	96.1	92.4	94.9	91.1	95.0	95.5	95.8	*	91.1	92.5	94.8	99.1	99.0	92.4	93.6	96.2	92.5	91.8	95.7	91.2	94.4	92.9	95.9	92.4
15	*Haloarcula marina* DT1^{T}	rrnA	91.2	95.2	91.1	95.9	92.5	95.2	92.8	95.1	94.6	91.1	*	93.3	95.2	91.1	90.9	95.9	92.3	91.8	95.2	95.2	91.8	93.6	92.5	95.5	91.7	95.1
		rrnB	95.0	91.0	94.1	92.6	95.7	91.1	93.3	92.3	95.0	92.5	93.3	*	92.5	95.0	94.8	92.4	95.4	94.7	91.1	90.5	95.2	89.9	89.9	91.5	96.1	91.2
16	*Haloarcula marismortui* ATCC 43049^{T}	rrnA	94.7	95.1	92.8	96.3	93.1	94.6	93.3	99.2	98.8	94.8	95.2	92.5	*	94.8	94.4	96.7	92.2	93.4	95.1	95.8	94.2	94.5	93.7	96.5	93.6	95.0
		rrnB	99.4	92.1	95.9	92.1	94.9	90.8	94.6	94.6	94.8	99.1	91.1	95.0	94.8	*	99.3	92.4	93.8	95.6	92.4	91.6	95.2	91.4	94.4	92.5	95.5	92.1
		rrnC	99.1	91.9	95.7	91.8	94.7	90.8	94.5	94.5	94.8	99.0	90.9	94.8	94.4	99.3	*	92.1	93.5	95.9	92.4	91.4	95.3	90.8	94.0	92.9	96.2	91.9
17	*Haloarcula nitratireducens* F27^{T}	rrnA	92.5	94.9	92.9	95.3	95.3	94.4	93.8	96.3	96.2	92.4	95.9	92.4	96.7	92.4	92.1	*	94.4	92.7	94.6	95.9	94.1	93.8	94.4	96.4	92.6	94.2
		rrnB	94.0	90.6	93.4	97.2	97.2	90.5	93.4	92.0	92.0	93.6	92.3	95.4	92.2	93.8	93.5	94.4	*	93.4	90.5	91.1	95.6	89.7	95.5	92.1	94.2	90.1
18	*Haloarcula onubensis* S3CR25-11^{T}	rrnA	95.7	94.3	97.5	93.4	95.4	91.2	95.0	93.6	93.7	96.2	91.8	94.7	93.4	95.6	95.9	92.7	93.4	*	94.4	91.9	95.3	90.7	94.6	93.3	96.2	94.3
		rrnB	92.5	98.0	93.2	94.6	91.7	94.3	92.7	95.1	95.0	92.5	95.2	91.1	95.1	92.4	92.4	94.6	90.5	94.4	*	96.3	92.0	93.8	91.8	95.4	92.4	98.1
19	*Haloarcula ordinaria* ZS-22-S1^{T}	rrnA	91.8	96.4	91.3	95.9	92.5	95.2	92.2	95.5	95.5	91.8	95.2	90.5	95.8	91.6	91.4	95.9	91.1	91.9	96.3	*	93.3	94.7	92.6	95.4	91.5	96.1
		rrnB	95.5	92.4	94.4	94.6	96.9	92.9	93.7	94.2	94.2	95.7	91.8	95.2	94.2	95.2	95.3	94.1	95.6	95.3	92.0	93.3	*	92.9	94.6	92.9	95.2	93.4
20	*Haloarcula pelagica* YJ-61-S^{T}	rrnA	91.4	94.0	90.1	93.8	91.0	95.4	91.2	94.4	94.2	91.2	93.6	89.9	94.5	91.4	90.8	93.8	89.7	90.7	93.8	94.7	92.9	*	92.7	93.3	91.2	93.7
		rrnB	94.3	92.7	93.8	94.7	95.9	91.2	93.3	93.5	93.5	94.4	92.5	89.9	93.7	94.4	94.0	94.4	95.5	94.6	91.8	92.6	94.6	92.7	*	92.9	95.3	91.9
21	*Haloarcula pellucida* CECT 7537^{T}	rrnA	92.7	95.0	93.2	92.9	92.9	94.5	95.1	96.5	96.3	92.9	95.5	91.5	96.5	92.5	92.9	96.4	92.1	93.3	95.4	95.4	92.9	93.3	92.9	*	94.9	95.0
		rrnB	95.7	92.7	95.9	95.5	95.5	91.4	92.9	93.9	94.0	95.9	91.7	96.1	93.6	95.5	96.2	92.6	94.2	96.2	92.4	91.5	95.2	91.2	95.3	94.9	*	92.6
22	*Haloarcula rara* SHR3^{T}	rrnA	92.1	98.8	93.3	94.4	91.8	93.8	92.2	95.1	95.2	92.4	95.1	91.2	95.0	92.1	91.9	94.2	90.1	94.3	98.1	96.1	93.4	93.7	91.9	95.0	92.6	*
		rrnB	96.0	93.5	97.4	94.6	91.6	91.2	94.8	93.4	93.5	96.2	91.5	94.8	93.1	96.1	96.1	94.4	90.5	98.3	93.7	92.6	94.6	90.9	94.1	94.5	91.4	94.4
23	*Haloarcula regularis* SYNS111^{T}	rrnA	90.8	94.8	90.6	96.1	92.7	94.4	92.0	94.2	94.1	91.1	96.3	91.4	94.4	90.8	90.5	95.6	91.9	91.2	94.9	97.0	92.8	93.5	93.1	94.3	90.8	94.4
		rrnB	94.6	90.8	93.7	93.5	95.8	91.0	94.3	92.8	92.8	95.0	91.9	95.3	92.9	94.6	94.6	93.1	95.2	94.1	90.8	91.6	97.9	90.8	96.1	91.9	94.4	90.4
24	*Haloarcula rubra* F13^{T}	rrnA	91.4	96.3	92.3	96.5	93.3	95.4	93.1	95.2	94.8	91.6	97.1	92.4	95.3	91.4	91.0	96.0	92.1	93.2	95.8	96.5	92.9	94.6	93.2	96.3	92.9	96.0
		rrnB	94.4	91.5	94.2	96.5	96.5	90.5	93.3	92.3	92.2	94.5	92.3	97.5	92.3	94.1	94.2	93.2	95.8	95.5	91.3	91.0	96.4	90.2	95.7	92.3	96.6	91.3
25	*Haloarcula rubripromontorii* SL3^{T}	rrnA	94.8	95.1	93.1	95.9	92.7	94.4	93.1	99.1	98.9	95.8	94.6	91.6	98.8	94.8	95.0	95.9	91.5	93.8	94.7	95.2	94.0	94.4	93.3	96.1	93.5	94.8
		rrnB	98.1	93.1	96.1	92.8	95.6	91.1	95.0	96.0	95.9	98.8	91.4	95.2	95.3	98.1	98.2	92.8	94.0	96.0	92.7	92.2	96.1	91.3	94.6	93.8	96.7	92.9
26	*Haloarcula salina* YGHS18^{T}	rrnA	93.5	93.8	92.7	96.1	94.0	93.7	93.3	96.6	96.6	93.9	94.2	93.4	96.0	93.3	93.6	96.0	93.1	92.7	93.2	94.4	94.7	93.6	94.0	95.1	93.4	93.1
		rrnB	96.2	91.9	94.9	93.3	95.9	91.3	94.9	93.8	93.8	96.6	91.6	95.8	93.1	96.0	96.3	93.2	94.3	94.9	91.2	91.6	96.1	91.3	95.2	92.7	95.7	91.4
27	*Haloarcula salinisoli* F24A^{T}	rrnA	91.5	98.5	92.7	91.3	91.3	93.5	94.9	94.7	94.8	91.8	94.9	90.7	94.6	91.5	91.3	94.6	90.2	93.8	97.9	95.7	91.5	93.5	91.3	94.8	92.1	99.1
		rrnB	96.1	94.5	97.5	93.1	95.2	91.5	95.3	93.8	94.0	96.5	92.1	94.8	93.5	95.9	96.2	92.9	93.1	99.0	94.2	92.2	95.2	91.3	94.6	93.6	96.5	94.7
28	*Halomicroarcula saliterrae* S1CR25-12^{T}	rrnA	91.9	98.0	92.4	95.6	92.4	94.9	92.4	95.5	95.4	91.9	95.4	90.6	95.8	92.0	91.7	95.7	90.9	93.0	98.0	97.5	92.7	94.3	92.3	95.3	91.7	97.8
		rrnB	96.3	92.6	97.0	92.6	94.8	91.3	95.2	93.1	93.0	96.3	91.0	94.9	92.9	96.2	96.2	92.5	93.9	97.4	92.7	91.5	94.7	90.4	94.3	92.9	95.9	92.7
29	*Haloarcula sebkhae* JCM 19018^{T}	rrnA	94.3	95.0	92.8	96.1	92.9	94.4	93.0	99.1	98.6	94.7	94.8	92.1	99.3	94.4	94.2	96.5	92.0	93.0	94.4	95.6	94.1	94.4	93.3	96.5	93.6	94.8
		rrnB	98.5	91.2	95.4	91.4	94.4	90.5	94.0	93.9	94.1	98.4	93.2	94.3	94.0	98.7	98.9	91.9	93.3	95.3	91.9	90.9	94.8	90.4	93.5	92.2	95.4	91.3
30	*Haloarcula sediminis* CK38^{T}	rrnA	92.7	98.8	93.5	94.6	91.8	94.2	92.4	95.4	95.4	93.0	95.3	90.9	95.0	92.6	92.3	94.5	90.4	93.8	97.3	95.8	91.6	94.2	92.2	94.8	92.4	98.6
		rrnB	96.3	93.8	97.9	92.6	94.8	91.2	94.9	94.0	94.0	96.5	91.8	94.6	93.7	96.3	95.9	92.5	93.6	96.9	92.9	91.4	94.2	90.8	94.4	93.8	96.4	93.9
31	*Haloarcula sinaiiensis* ATCC 33800^{T}	rrnA	98.7	91.6	95.4	91.4	94.3	90.2	94.0	93.9	94.2	98.4	90.3	94.7	94.0	98.9	98.8	91.7	93.2	95.5	91.8	91.0	94.9	90.6	93.9	91.8	95.3	91.6
		rrnB	97.1	93.5	94.5	93.6	93.9	91.6	93.8	95.9	95.9	96.7	92.3	93.9	96.1	97.3	96.9	93.9	92.8	94.4	93.8	93.1	94.4	91.8	93.2	93.5	94.2	93.3
32	*Haloarcula taiwanensis* Taiwanensis^{T}	rrn1	94.4	95.3	93.3	96.0	92.8	94.4	92.9	99.5	99.3	95.3	94.6	92.0	98.9	94.4	94.8	96.0	91.7	93.5	94.8	95.2	94.0	94.1	93.1	96.7	94.1	94.9
		rrn2	98.8	92.6	96.2	92.3	95.0	91.2	94.8	95.0	95.3	99.5	90.6	94.6	94.4	98.9	99.3	92.1	93.6	96.2	92.5	91.6	95.5	90.9	94.1	93.0	96.2	92.3
33	*Haloarcula terrestris* S1AR25-5A^{T}	rrnA	94.5	95.3	93.1	96.2	93.0	94.5	93.1	99.5	99.1	95.2	94.9	92.1	99.5	94.6	94.2	96.3	91.8	93.6	94.9	95.6	94.4	94.6	93.7	96.3	93.7	95.0
		rrnB	99.7	90.6	95.9	91.6	95.9	89.5	94.7	93.6	93.9	99.3	89.7	95.9	93.3	99.7	99.1	91.7	94.6	95.8	90.3	89.3	96.0	90.2	95.0	91.1	96.3	90.5
34	*Haloarcula vallismortis* ATCC 29715^{T}	rrnA	94.0	93.8	93.1	95.9	93.0	94.0	93.1	98.8	98.5	94.8	94.5	93.5	98.6	96.8	94.4	95.9	91.8	93.9	94.4	95.1	94.4	94.0	93.5	96.1	94.1	94.7
		rrnB	98.8	92.2	96.0	92.1	94.9	90.7	94.7	94.6	94.8	99.0	91.6	95.0	94.4	99.0	99.3	92.0	93.7	96.2	92.5	91.6	95.4	91.0	94.1	92.9	95.9	92.1

No.	16S rRNA gene sequence similarity (%)		22	23		24		25		26		27		28		29		30		31		32		33		34	
			rrnB	rrnA	rrnB	rrnA	rrnB	rrnA	rrnB	rrnA	rrnB	rrnA	rrnB	rrnA	rrnB	rrnA	rrnB	rrnA	rrnB	rrnA	rrnB	rrn1	rrn2	rrnA	rrnB	rrnA	rrnB
1	Strain GH36^{T}	rrnA	91.0	92.9	90.5	94.1	90.2	94.0	91.0	93.5	91.2	93.2	90.9	93.9	90.6	93.7	90.2	93.8	90.8	90.5	91.4	93.8	90.6	94.3	89.7	94.0	91.0
		rrnB	93.8	93.6	96.9	93.3	95.9	93.2	94.5	94.0	95.1	91.3	93.9	92.1	94.3	92.9	93.6	92.0	94.1	94.0	93.3	93.2	94.1	93.6	95.1	93.6	94.2
2	*Haloarcula amylolytica* JCM 13557^{T}	rrnA	93.0	94.1	92.9	95.0	92.2	98.9	95.3	96.0	93.1	94.3	93.4	95.2	92.7	98.9	93.8	94.8	93.4	93.9	95.7	98.8	94.5	99.3	93.3	98.6	94.0
		rrnB	95.4	90.8	94.6	91.9	94.9	94.8	98.8	94.1	96.8	91.9	95.7	92.4	95.4	94.8	98.0	92.1	95.7	97.8	96.9	95.5	98.6	95.0	99.3	94.8	98.0
		rrnC	95.5	90.8	94.6	91.3	94.2	94.7	98.0	93.6	96.3	91.0	95.7	91.7	96.2	94.1	98.6	92.3	95.7	98.4	96.9	94.7	99.2	94.3	99.1	93.9	98.7
3	*Haloarcula amylovorans* LR21^{T}	rrnA	92.3	95.8	93.4	95.7	93.2	95.9	92.3	96.0	93.1	94.0	92.9	95.1	92.7	95.0	91.8	94.9	92.9	91.9	93.7	95.5	92.2	95.9	92.1	95.5	92.4
		rrnB	91.7	92.8	96.3	92.9	96.4	93.1	95.2	93.9	95.8	91.1	95.0	92.0	94.8	92.9	94.4	92.2	95.1	94.6	93.8	92.7	95.1	93.1	96.1	92.9	95.0
4	*Haloarcula argentinensis* DSM 12282^{T}	rrnA	93.1	93.8	92.7	95.0	92.2	98.6	95.6	96.4	93.7	94.2	93.4	95.2	92.7	99.2	98.0	94.5	93.5	94.0	95.7	99.2	95.0	99.0	93.6	94.8	98.5
		rrnB	95.9	91.0	94.8	91.7	94.4	95.1	98.2	93.8	96.3	91.4	95.9	92.1	96.3	94.8	98.6	92.4	96.0	98.7	97.1	94.8	99.2	94.7	99.4	94.5	99.1
5	*Haloarcula brevis* DT43^{T}	rrnA	94.0	94.6	93.3	94.9	92.4	98.4	95.5	96.7	94.0	94.5	93.9	95.6	94.0	98.1	94.5	95.1	93.8	94.6	96.7	98.7	95.5	98.9	94.0	98.2	95.2
		rrnB	95.9	91.6	95.3	91.9	94.9	95.0	98.5	94.2	96.9	91.8	95.8	92.4	96.5	94.3	97.9	92.7	96.1	97.9	97.3	95.0	98.8	95.2	98.9	94.8	98.5
6	*Haloarcula californiae* ATCC 33799^{T}	rrnA	96.2	90.6	94.5	90.9	94.2	94.9	98.3	93.0	95.8	91.7	95.9	91.8	96.2	93.8	98.7	92.5	96.3	99.0	97.1	94.5	99.0	94.2	99.2	94.1	99.3
		rrnB	93.2	94.1	92.6	95.1	92.1	98.8	95.2	96.1	93.2	94.7	93.1	95.6	92.8	99.1	94.1	94.9	93.6	93.9	95.9	98.8	94.3	99.5	93.0	98.6	94.3
7	*Haloarcula halophila* DFY41^{T}	rrnA	91.0	92.9	90.5	94.2	90.1	94.0	91.0	93.5	91.2	93.2	91.3	93.9	90.5	94.1	94.1	93.8	90.7	90.5	91.4	93.8	90.6	94.3	89.7	94.0	91.0
		rrnB	93.8	93.5	96.8	93.4	95.8	93.1	94.4	93.9	95.0	91.2	94.3	92.1	94.3	93.3	93.6	91.9	94.0	93.9	93.3	93.1	94.0	93.6	95.0	93.6	94.1
8	*Haloarcula halobia* XH51^{T}	rrnA	90.1	99.6	94.2	96.0	91.9	93.9	90.7	94.0	91.2	93.9	91.0	95.9	90.7	94.1	90.1	94.6	90.4	90.1	92.0	93.6	90.6	94.0	89.5	93.6	90.8
		rrnB	93.0	94.4	99.5	92.3	95.8	93.0	94.6	93.5	94.8	89.9	93.9	91.4	93.7	93.1	94.2	90.7	93.3	94.3	93.7	92.6	94.6	92.9	95.6	93.1	94.8
9	*Haloarcula hispanica* ATCC 33960^{T}	rrn1	95.6	91.0	94.8	91.7	94.7	96.4	98.4	94.0	96.7	91.7	96.1	92.6	95.9	95.0	98.3	92.7	96.3	97.9	97.6	95.0	98.8	94.9	99.6	94.8	98.8
		rrn2	95.9	91.0	94.8	91.4	94.4	96.8	96.5	93.7	96.4	91.4	96.3	92.3	96.2	94.7	98.6	92.4	96.0	96.3	95.0	94.8	99.1	94.6	99.6	94.5	99.1
		rrn3	93.1	94.3	92.9	95.3	92.3	99.1	95.6	96.3	93.5	94.5	93.7	95.6	92.9	99.4	93.8	95.0	93.8	93.7	95.6	99.2	94.7	99.7	93.3	98.9	94.3
10	*Haloarcula japonica* DSM 6131^{T}	rrnA	93.3	93.7	92.6	94.8	92.0	98.7	95.4	96.4	93.6	94.1	93.6	94.9	93.1	98.7	94.0	94.8	93.8	94.3	95.9	98.9	94.9	99.1	94.0	98.4	94.8
		rrnB	95.7	90.8	94.6	91.8	94.8	95.1	98.6	94.2	96.9	91.7	95.8	92.1	95.9	94.6	98.3	92.5	96.1	98.2	96.7	95.4	99.1	95.0	99.5	94.6	98.4
		rrnC	96.0	90.8	94.6	91.4	94.4	94.8	98.1	93.5	96.2	91.5	96.1	91.9	96.3	94.3	98.5	92.7	96.3	98.7	97.1	94.4	98.8	94.5	99.7	94.0	98.8
11	*Haloarcula laminariae* LYG-108^{T}	rrnA	93.5	94.8	90.8	96.3	91.5	95.1	93.1	93.8	91.9	98.5	94.5	98.0	92.6	95.0	91.2	98.8	93.8	91.6	93.5	95.3	92.6	95.3	90.6	93.8	92.2
		rrnB	97.4	90.6	93.7	92.3	94.2	93.1	96.1	92.7	94.9	92.7	97.5	92.4	97.0	92.8	95.4	93.5	97.9	95.4	94.5	93.3	96.2	93.1	95.9	93.1	96.0
12	*Haloarcula limicola* YGHS32^{T}	rrnA	94.6	96.1	93.5	96.5	96.5	95.9	92.8	96.1	93.3	91.3	93.1	95.6	92.6	96.1	91.4	94.6	92.6	91.4	93.6	96.0	92.3	96.2	91.6	95.9	92.1
		rrnB	91.6	92.7	95.8	93.3	96.5	92.7	95.6	94.0	95.9	91.3	95.2	92.4	94.8	92.9	94.4	91.8	94.8	94.3	93.9	92.8	95.0	93.0	95.9	93.0	94.9
13	*Haloarcula litorea* GDY20^{T}	rrnA	91.2	94.4	91.0	95.4	90.5	94.4	91.1	93.7	91.3	93.5	91.5	94.9	91.3	94.4	90.5	94.2	91.2	90.2	91.6	94.4	91.2	94.5	89.5	94.0	90.7
		rrnB	94.8	92.0	94.3	93.1	93.3	93.1	95.0	93.3	94.9	94.9	95.3	92.4	95.2	93.0	94.0	92.4	94.9	94.0	93.8	92.9	94.8	93.1	94.7	93.1	94.7
14	*Haloarcula mannanilytica* MD-130^{T}	rrn1	93.4	94.2	92.8	95.2	92.3	99.1	96.0	96.6	93.8	94.7	93.8	95.5	93.1	99.1	93.9	95.4	94.0	93.9	95.9	99.5	95.0	99.5	93.6	98.8	94.6
		rrn2	93.5	94.1	92.8	94.8	92.2	98.9	95.9	96.6	93.8	94.8	94.0	95.4	93.0	98.6	94.1	95.4	94.0	94.2	95.9	99.3	95.3	99.1	93.9	98.5	94.8
		rrn3	96.2	91.1	95.0	91.6	94.5	95.8	98.8	93.9	96.6	91.8	96.5	91.9	96.3	94.7	98.4	93.0	96.5	98.4	96.7	95.3	99.5	95.2	99.3	94.8	99.0
15	*Haloarcula marina* DT1^{T}	rrnA	91.5	96.3	91.9	97.1	92.3	94.6	91.4	94.2	91.6	94.9	92.1	95.4	91.0	94.8	93.2	95.3	91.8	90.3	92.3	94.6	90.6	94.9	89.7	94.5	91.6
		rrnB	94.8	91.4	95.3	92.4	97.5	91.6	95.2	93.4	95.8	90.7	94.8	90.6	94.9	92.1	94.3	90.9	94.6	94.7	93.9	92.0	94.6	92.1	95.9	93.5	95.0
16	*Haloarcula marismortui* ATCC 43049^{T}	rrnA	93.1	94.4	92.9	95.3	92.3	98.8	95.3	96.0	93.1	94.6	93.5	95.8	92.9	99.3	94.0	95.0	93.7	94.0	96.1	98.9	94.4	99.5	93.3	98.6	94.4
		rrnB	96.1	90.8	94.6	91.4	94.1	94.8	98.1	93.3	96.0	91.5	95.9	92.0	96.2	94.4	98.7	92.6	96.3	98.9	97.3	94.4	98.9	94.6	99.7	96.8	99.0
		rrnC	96.1	90.5	94.6	91.0	94.2	95.0	98.2	93.6	96.3	91.3	96.2	91.7	96.2	94.2	98.9	92.3	95.9	98.8	96.9	94.8	99.3	94.2	99.1	94.4	99.3
17	*Haloarcula nitratireducens* F27^{T}	rrnA	94.4	95.6	93.1	96.0	93.2	95.9	92.8	96.0	93.2	94.6	92.9	95.7	92.5	96.5	91.9	94.5	92.5	91.7	93.9	96.0	92.1	96.3	91.7	95.9	92.0
		rrnB	90.5	91.9	95.2	92.1	95.8	91.5	94.0	93.1	94.3	90.2	93.1	90.9	93.9	92.0	93.3	90.4	93.6	93.2	92.8	91.7	93.6	91.8	94.6	91.8	93.7
18	*Haloarcula onubensis* S3CR25-11^{T}	rrnA	98.3	91.2	94.1	93.2	95.5	93.8	96.0	92.7	94.9	93.8	99.0	93.0	97.4	93.0	95.3	93.8	96.9	95.5	94.4	93.5	96.2	93.6	95.8	93.9	96.2
		rrnB	93.7	94.9	90.8	95.8	91.3	94.7	92.7	93.2	91.2	97.9	94.2	98.0	92.7	94.4	91.9	97.3	92.9	91.8	93.8	94.8	92.5	94.9	90.3	94.4	92.5
19	*Haloarcula ordinaria* ZS-22-S1^{T}	rrnA	92.6	97.0	91.6	96.5	91.0	95.2	92.2	94.4	91.6	95.7	92.2	97.5	91.5	95.6	90.9	95.8	91.4	91.0	93.1	95.2	91.6	95.6	89.3	95.1	91.6
		rrnB	94.6	92.8	97.9	92.9	96.4	94.0	96.1	94.7	96.1	91.5	95.2	92.7	94.7	94.1	94.8	91.6	94.2	94.9	94.4	94.0	95.5	94.4	96.0	94.4	95.4
20	*Haloarcula pelagica* YJ-61-S^{T}	rrnA	90.9	93.5	90.8	94.6	90.2	94.4	91.3	93.6	91.3	93.5	91.3	94.3	90.4	94.4	90.4	94.2	90.8	90.6	91.8	94.1	90.9	94.6	90.2	94.0	91.0
		rrnB	94.1	93.1	96.1	93.2	95.7	93.3	94.6	94.0	95.2	91.3	94.6	92.3	94.3	93.3	93.5	92.2	94.4	93.9	93.2	93.1	94.1	93.7	95.0	93.5	94.1
21	*Haloarcula pellucida* CECT 7537^{T}	rrnA	94.5	94.3	91.9	96.3	92.3	96.1	93.8	95.1	92.7	94.8	93.6	95.3	92.9	96.5	92.2	94.8	93.8	91.8	93.5	96.7	93.0	96.3	91.1	96.1	92.9
		rrnB	91.4	90.8	94.4	92.9	96.6	93.5	96.7	93.4	95.7	92.1	96.5	91.7	95.9	93.6	95.4	92.4	96.4	95.3	94.2	94.1	96.2	93.7	96.3	94.1	95.9
22	*Haloarcula rara* SHR3^{T}	rrnA	94.4	94.4	90.4	96.0	91.3	94.8	92.9	93.1	91.4	99.1	94.7	97.8	92.7	94.8	91.3	98.6	93.9	91.6	93.3	94.9	92.3	95.0	90.5	94.7	92.1
		rrnB	*	90.2	93.1	92.6	94.3	93.6	96.1	92.5	94.7	93.6	98.8	92.6	98.1	93.1	95.5	94.0	98.0	95.7	94.2	93.3	96.2	93.3	96.0	93.3	96.2
23	*Haloarcula regularis* SYNS111^{T}	rrnA	90.2	*	94.4	95.9	92.0	94.3	90.9	94.4	91.5	94.1	90.6	95.7	90.8	93.6	90.0	94.8	90.5	90.1	92.0	94.0	90.9	94.4	89.9	94.0	90.8
		rrnB	93.1	94.4	*	92.1	96.0	93.0	94.7	93.5	94.9	89.9	93.5	91.1	93.9	92.2	94.1	90.7	93.3	94.3	93.5	92.6	94.8	92.9	95.8	93.1	94.7
24	*Haloarcula rubra* F13^{T}	rrnA	92.6	95.9	92.1	*	93.4	94.6	91.9	94.2	91.9	96.4	93.3	96.4	92.6	95.2	90.8	95.8	92.4	90.4	92.3	94.8	91.2	95.1	90.0	94.7	91.2
		rrnB	94.3	92.0	96.0	93.4	*	92.0	95.0	93.4	95.6	91.6	95.4	90.9	95.1	92.2	93.9	91.0	94.1	94.0	93.3	92.2	94.4	92.4	95.9	92.4	94.3
25	*Haloarcula rubripromontorii* SL3^{T}	rrnA	93.6	94.3	93.0	94.6	92.0	*	95.6	96.1	93.3	94.4	93.6	95.1	93.4	98.4	94.3	95.6	94.1	94.3	95.4	99.1	95.5	99.1	93.1	98.7	95.0
		rrnB	96.1	90.9	94.7	91.9	95.0	95.6	*	94.1	96.8	92.4	95.9	92.7	96.0	94.8	97.7	92.6	96.6	97.6	96.6	96.0	98.8	95.7	99.0	95.4	98.2
26	*Haloarcula salina* YGHS18^{T}	rrnA	92.5	94.4	93.5	94.2	93.4	96.1	94.1	*	97.0	93.3	92.9	93.7	92.4	96.3	93.3	93.8	92.9	92.7	94.4	96.8	94.1	96.5	93.8	96.4	93.5
		rrnB	94.7	91.5	94.9	91.9	95.6	93.3	96.8	97.0	*	91.4	95.0	91.3	94.9	93.4	96.1	91.7	95.1	95.5	94.6	94.0	96.8	93.6	97.9	93.6	96.2
27	*Haloarcula salinisoli* F24A^{T}	rrnA	93.6	94.1	89.9	96.4	91.6	94.4	92.4	92.7	90.7	*	94.7	97.6	92.7	95.1	91.0	97.9	93.1	90.9	92.7	94.5	91.7	94.7	90.4	94.3	91.6
		rrnB	98.8	90.6	93.5	93.3	95.4	93.6	95.9	92.9	95.0	94.7	*	93.0	97.7	93.5	95.5	93.9	97.0	95.2	94.0	93.3	96.1	93.3	95.8	94.0	96.5
28	*Halomicroarcula saliterrae* S1CR25-12^{T}	rrnA	92.6	95.7	91.1	96.4	90.9	95.1	92.7	93.7	91.3	97.6	93.0	*	92.8	95.1	91.4	97.3	92.6	91.3	93.5	95.2	91.9	95.6	89.9	95.0	91.9
		rrnB	98.1	90.8	93.9	92.6	95.1	93.4	96.0	92.4	94.9	92.7	97.7	92.8	*	92.2	95.8	92.9	97.1	95.9	94.6	92.9	96.5	93.0	96.0	92.8	96.4
29	*Haloarcula sebkhae* JCM 19018^{T}	rrnA	93.1	93.6	92.2	95.2	92.2	98.4	94.8	96.3	93.4	95.1	93.5	95.1	92.2	*	94.0	94.2	92.9	93.3	95.3	98.8	94.2	99.0	92.7	99.0	94.5
		rrnB	95.5	90.0	94.1	90.8	93.9	94.3	97.7	93.3	96.1	91.0	95.5	91.4	95.8	94.0	*	91.5	95.6	98.3	96.5	94.2	98.8	94.0	98.3	93.6	98.3
30	*Haloarcula sediminis* CK38^{T}	rrnA	94.0	94.8	90.7	95.8	91.0	95.6	92.6	93.8	91.7	97.9	93.9	97.3	92.9	94.2	91.5	*	94.9	91.8	93.1	94.9	92.8	95.2	90.7	94.6	92.5
		rrnB	98.0	90.5	93.3	92.4	94.1	94.1	96.6	92.9	95.1	93.1	97.0	92.6	97.1	92.9	95.6	94.9	*	95.6	94.2	93.7	96.3	94.0	96.5	93.6	96.2
31	*Haloarcula sinaiiensis* ATCC 33800^{T}	rrnA	95.7	90.1	94.3	90.4	94.0	94.3	97.6	92.7	95.5	90.9	95.2	91.3	95.9	93.3	98.3	91.8	95.6	*	97.4	93.8	98.3	93.8	99.1	93.7	98.6
		rrnB	94.2	92.0	93.5	92.3	93.3	95.4	96.6	94.4	94.6	92.7	94.0	93.5	94.6	95.3	96.5	93.1	94.2	97.4	*	95.6	96.7	95.7	96.9	95.3	96.9
32	*Haloarcula taiwanensis* Taiwanensis^{T}	rrn1	93.3	94.0	92.6	94.8	92.2	99.1	96.0	96.8	94.0	94.5	93.3	95.2	92.9	98.8	94.2	94.9	93.7	93.8	95.6	*	95.5	99.2	93.4	98.7	94.6
		rrn2	96.2	90.9	94.8	91.2	94.4	95.5	98.8	94.1	96.8	91.7	96.1	91.9	96.5	94.2	98.8	92.8	96.3	98.3	96.7	95.5	*	94.7	99.1	94.6	99.1
33	*Haloarcula terrestris* S1AR25-5A^{T}	rrnA	93.3	94.4	92.9	95.1	92.4	99.1	95.7	96.5	93.6	94.7	93.3	95.6	93.0	99.0	94.0	95.2	94.0	93.8	95.7	99.2	94.7	*	93.7	98.9	94.3
		rrnB	96.0	89.9	95.8	90.0	95.9	93.1	99.0	93.8	97.9	90.4	95.8	89.9	96.0	92.7	98.3	90.7	96.5	99.1	96.9	93.4	99.1	93.7	*	93.0	98.9
34	*Haloarcula vallismortis* ATCC 29715^{T}	rrnA	93.3	94.0	93.1	94.7	92.4	98.7	95.4	96.4	93.6	94.3	94.0	95.0	92.8	99.0	93.6	94.6	93.6	93.7	95.3	98.7	94.6	98.9	93.0	*	94.6
		rrnB	96.2	90.8	94.7	91.2	94.3	95.0	98.2	93.5	96.2	91.6	96.5	91.9	96.4	94.5	98.3	92.5	96.2	98.6	96.9	94.6	99.1	94.3	98.9	94.6	*

Table S2. *rpoB′* gene sequence similarities among strain GH36T and the current species of the genus *Haloarcula*.

No.	*rpoB′* gene sequence similarity (%)	1	2	3	4	5	6	7	8	9	10	11	12	13	14	15	16	17	18	19	20	21	22	23	24	25	26	27	28	29	30	31	32	33	34
1	Strain GH36T	*	90.0	89.1	88.3	90.0	89.3	97.6	89.8	89.4	89.3	88.5	89.9	89.7	89.4	88.6	89.1	90.2	88.8	89.1	92.4	89.1	89.2	89.9	90.4	89.2	89.7	88.8	89.8	88.5	88.6	89.3	89.3	89.4	89.2
2	*Haloarcula amylolytica* JCM 13557T	90.0	*	90.3	94.3	95.6	95.1	90.3	91.3	96.2	95.1	91.2	91.3	89.1	95.0	90.4	95.3	91.8	90.3	91.4	91.0	91.6	90.6	91.4	91.8	94.9	92.2	91.0	91.5	95.1	91.1	95.4	95.6	95.3	95.2
3	*Haloarcula amylovorans* LR21T	89.1	90.3	*	89.3	91.5	89.9	89.2	89.8	90.2	90.3	90.0	95.2	88.9	91.4	90.8	89.6	94.6	89.4	89.9	90.3	91.1	89.7	89.7	92.1	91.0	91.0	90.2	90.4	89.8	90.3	89.8	90.9	90.2	91.0
4	*Haloarcula argentinensis* DSM 12282T	88.3	94.3	89.3	*	94.2	96.1	88.3	89.8	94.8	95.3	90.0	90.4	87.6	93.9	89.4	96.2	91.1	88.6	90.1	89.8	90.4	89.4	89.8	91.0	94.0	91.7	89.9	90.0	95.6	89.4	96.2	94.0	94.0	94.4
5	*Haloarcula brevis* DT43T	90.0	95.6	91.5	94.2	*	95.0	90.2	91.5	95.2	95.8	91.6	92.6	90.1	94.8	91.3	94.7	93.2	91.1	91.4	91.8	92.5	91.6	91.6	92.6	95.8	93.3	91.7	92.2	94.9	91.8	95.0	95.9	94.5	94.7
6	*Haloarcula californiae* ATCC 33799T	89.3	95.1	89.9	96.1	95.0	*	89.2	90.4	96.0	96.7	90.4	90.9	87.9	94.4	89.7	98.9	91.6	89.3	90.8	90.3	91.0	89.6	90.3	91.1	94.8	92.0	90.2	90.4	95.5	89.9	99.4	94.7	95.1	94.7
7	*Haloarcula halophila* DFY41T	97.6	90.3	89.2	88.3	90.2	89.2	*	90.0	89.3	89.3	88.5	90.0	89.4	89.8	88.3	89.1	90.2	88.8	89.0	92.5	89.3	89.4	90.3	90.6	89.6	89.4	89.3	89.8	88.8	88.7	89.3	89.3	89.6	89.3
8	*Haloarcula halobia* XH51T	89.8	91.3	89.8	89.8	91.5	90.4	90.0	*	90.6	90.7	90.6	91.5	89.8	90.4	90.0	90.3	91.7	90.5	94.9	90.2	91.1	90.9	94.9	90.8	90.8	91.3	90.5	91.7	90.1	90.6	90.5	90.4	90.4	90.5
9	*Haloarcula hispanica* ATCC 33960T	89.4	96.2	90.2	94.8	95.2	96.0	89.3	90.6	*	95.7	90.7	91.2	88.6	94.3	90.0	96.5	91.6	89.8	90.7	90.4	91.5	89.7	90.5	91.8	95.2	92.0	90.0	90.6	95.6	90.8	96.3	95.9	94.6	94.7
10	*Haloarcula japonica* DSM 6131T	89.3	95.1	90.3	95.3	95.8	96.7	89.3	90.7	95.7	*	90.9	91.3	88.8	94.8	90.6	96.1	91.6	89.7	90.7	90.9	91.2	90.2	90.5	91.9	95.1	92.5	90.6	90.8	95.8	90.6	96.4	94.8	95.0	94.7
11	*Haloarcula laminariae* LYG-108T	88.5	91.2	90.0	90.0	91.6	90.4	88.5	90.6	90.7	90.9	*	91.0	89.7	90.5	89.6	90.2	91.2	92.3	90.0	89.6	90.5	93.5	90.4	90.7	91.5	91.6	93.4	94.0	90.2	93.9	90.2	90.9	90.9	90.5
12	*Haloarcula limicola* YGHS32T	89.9	91.3	95.2	90.4	92.6	90.9	90.0	91.5	91.2	91.3	91.0	*	91.4	91.6	91.2	90.9	96.6	91.2	91.1	91.2	91.7	90.5	91.8	92.5	92.1	92.7	90.6	91.6	90.5	91.7	91.1	91.8	90.8	91.3
13	*Haloarcula litorea* GDY20T	89.7	89.1	88.9	87.6	90.1	87.9	89.4	89.8	88.6	88.8	89.7	91.4	*	88.8	89.2	88.0	90.6	89.8	89.2	90.4	89.9	90.1	90.2	90.6	89.2	90.5	89.7	90.1	88.2	89.6	88.1	88.9	88.5	88.6
14	*Haloarcula mannanilytica* MD-130T	89.4	95.0	91.4	93.9	94.8	94.4	89.8	90.4	94.3	94.8	90.5	91.6	88.8	*	89.9	94.4	92.2	90.2	90.6	91.0	91.4	90.4	90.9	91.6	95.1	92.3	91.0	91.2	94.1	90.5	94.8	95.3	94.8	95.7
15	*Haloarcula marina* DT1T	88.6	90.4	90.8	89.4	91.3	89.7	88.3	90.0	90.0	90.6	89.6	91.2	89.2	89.9	*	89.4	92.2	90.2	90.6	89.9	92.8	89.4	90.4	93.5	90.5	91.1	89.6	90.8	90.3	88.9	89.6	90.4	89.9	90.3
16	*Haloarcula marismortui* ATCC 43049T	89.1	95.3	89.6	96.2	94.7	98.9	89.1	90.3	96.5	96.1	90.2	90.9	88.0	94.4	89.4	*	91.6	89.1	90.7	90.3	90.9	89.8	90.3	91.1	95.1	91.7	90.4	90.4	95.7	90.0	99.5	94.6	94.9	94.8
17	*Haloarcula nitratireducens* F27T	90.2	91.8	94.6	91.1	93.2	91.6	90.2	91.7	91.6	91.6	91.2	96.6	90.6	92.2	92.2	91.6	*	91.0	91.6	91.7	92.6	90.5	91.9	93.2	92.7	92.8	90.8	91.2	91.6	91.7	91.6	92.5	91.7	92.4
18	*Haloarcula onubensis* S3CR25-11T	88.8	90.3	89.4	88.6	91.1	89.3	88.8	90.5	89.8	89.7	92.3	91.2	89.8	90.2	90.2	89.1	91.0	*	90.1	89.9	90.8	92.4	90.2	90.5	90.3	92.0	92.7	92.7	88.9	92.0	89.2	90.8	89.3	89.9
19	*Haloarcula ordinaria* ZS-22-S1T	89.1	91.4	89.9	90.1	91.4	90.8	89.0	94.9	90.7	90.7	90.0	91.1	89.2	90.6	90.6	90.7	91.6	90.1	*	90.2	90.3	90.3	94.5	90.5	91.0	91.5	90.3	90.9	90.1	90.2	90.9	91.1	90.4	90.6
20	*Haloarcula pelagica* YJ-61-ST	92.4	91.0	90.3	89.8	91.8	90.3	92.5	90.2	90.4	90.9	89.6	91.2	90.4	91.0	89.9	90.3	91.7	89.9	90.2	*	90.5	90.2	90.5	91.6	90.9	91.8	90.4	90.3	89.9	89.8	90.3	90.0	89.8	90.2
21	*Haloarcula pellucida* CECT 7537T	89.1	91.6	91.1	90.4	92.5	91.0	89.3	91.1	91.5	91.2	90.5	91.7	89.9	91.4	92.8	90.9	92.6	90.8	90.3	90.5	*	89.8	90.6	95.0	92.2	91.6	90.7	91.4	90.9	90.6	90.8	91.5	91.8	90.9
22	*Haloarcula rara* SHR3T	89.2	90.6	89.7	89.4	91.6	89.6	89.4	90.9	89.7	90.2	93.5	90.5	90.1	90.4	89.4	89.8	90.5	92.4	90.3	90.2	89.8	*	91.1	90.6	90.5	90.8	95.0	93.4	89.9	92.7	89.6	90.4	90.1	90.4
23	*Haloarcula regularis* SYNS111T	89.9	91.4	89.7	89.8	91.6	90.3	90.3	94.9	90.5	90.5	90.4	91.8	90.2	90.9	90.4	90.3	91.9	90.2	94.5	90.5	90.6	91.1	*	91.5	91.5	92.3	90.7	91.6	90.1	90.6	90.4	91.1	90.4	90.3
24	*Haloarcula rubra* F13T	90.4	91.8	92.1	91.0	92.6	91.1	90.6	90.8	91.8	91.9	90.7	92.5	90.6	91.6	93.5	91.1	93.2	90.5	90.5	91.6	95.0	90.6	91.5	*	92.7	91.9	91.3	91.7	91.5	90.3	91.1	91.8	91.7	91.4
25	*Haloarcula rubripromontorii* SL3T	89.2	94.9	91.0	94.0	95.8	94.8	89.6	90.8	95.2	95.1	91.5	92.1	89.2	95.1	90.5	95.1	92.7	90.3	91.0	90.9	92.2	90.5	91.5	92.7	*	93.1	91.5	91.5	94.6	91.1	94.9	94.9	94.9	95.1
26	*Haloarcula salina* YGHS18T	89.7	92.2	91.0	91.7	93.3	92.0	89.4	91.3	92.0	92.5	91.6	92.7	90.5	92.3	91.1	91.7	92.8	92.0	91.5	91.8	91.6	90.8	92.3	91.9	93.1	*	91.3	92.0	91.3	91.0	91.8	92.3	91.4	92.0
27	*Haloarcula salinisoli* F24AT	88.8	91.0	90.2	89.9	91.7	90.2	89.3	90.5	90.0	90.6	93.4	90.6	89.7	91.0	89.6	90.4	90.8	92.7	90.3	90.4	90.7	95.0	90.7	91.3	91.5	91.3	*	93.3	90.7	93.2	90.3	91.5	90.8	90.8
28	*Haloarcula saliterrae* S1CR25-12T	89.8	91.5	90.4	90.0	92.2	90.4	89.8	91.7	90.6	90.8	94.0	91.6	90.1	91.2	90.8	90.4	91.2	92.7	90.9	90.3	91.4	93.4	91.6	91.7	91.5	92.0	93.3	*	90.0	92.9	90.4	91.4	90.5	90.5
29	*Haloarcula sebkhae* JCM 19018T	88.5	95.1	89.8	95.6	94.9	95.5	88.8	90.1	95.6	95.8	90.2	90.5	88.2	94.1	90.3	95.7	91.6	88.9	90.1	89.9	90.9	89.9	90.1	91.5	94.6	91.3	90.7	90.0	*	89.8	95.6	94.7	94.6	94.6
30	*Haloarcula sediminis* CK38T	88.6	91.1	90.3	89.4	91.8	89.9	88.7	90.6	90.8	90.6	93.9	91.7	89.6	90.5	88.9	90.0	91.7	92.0	90.2	89.8	90.6	92.7	90.6	90.3	91.1	91.0	93.2	92.9	89.8	*	90.0	91.2	90.7	90.5
31	*Haloarcula sinaiiensis* ATCC 33800T	89.3	95.4	89.8	96.2	95.0	99.4	89.3	90.5	96.3	96.4	90.2	91.1	88.1	94.8	89.6	99.5	91.6	89.2	90.9	90.3	90.8	89.6	90.4	91.1	94.9	91.8	90.3	90.4	95.6	90.0	*	94.8	95.2	94.9
32	*Haloarcula taiwanensis* TaiwanensisT	89.3	95.6	90.9	94.0	95.9	94.7	89.3	90.4	95.9	94.8	90.9	91.8	88.9	95.3	90.4	94.6	92.5	90.8	91.1	90.0	91.5	90.4	91.1	91.8	94.9	92.3	91.5	91.4	94.7	91.2	94.8	*	94.8	95.2
33	*Haloarcula terrestris* S1AR25-5AT	89.4	95.3	90.2	94.0	94.5	95.1	89.6	90.4	94.6	95.0	90.9	90.8	88.5	94.8	89.9	94.9	91.7	89.3	90.4	89.8	91.8	90.1	90.4	91.7	94.9	91.4	90.8	90.5	94.6	90.7	95.2	94.8	*	95.1
34	*Haloarcula vallismortis* ATCC 29715T	89.2	95.2	91.0	94.4	94.7	94.7	89.3	90.5	94.7	94.7	90.5	91.3	88.6	95.7	90.3	94.8	92.4	89.9	90.6	90.2	90.9	90.4	90.3	91.4	95.1	92.0	90.8	90.5	94.6	90.5	94.9	95.2	95.1	*

Table S3. Pairwise ANI values among strain GH36^T and the current species of the genus *Haloarcula*. Values exceeding the species-level threshold of 96% are highlighted in yellow.

No.	ANI (%)	1	2	3	4	5	6	7	8	9	10	11	12	13	14	15	16	17	18	19	20	21	22	23	24	25	26	27	28	29	30	31	32	33	34
1	Strain GH36^T	*	79.4	78.8	78.9	79.3	77.4	95.5	79.0	79.3	79.1	79.9	79.9	79.8	79.0	79.6	78.6	80.3	79.3	78.9	86.5	79.9	78.5	78.9	80.3	78.1	80.0	79.4	79.2	78.9	78.5	77.5	78.0	79.7	78.7
2	*Haloarcula amylolytica* JCM 13557^T	79.4	*	75.7	89.2	88.4	90.3	76.7	77.7	94.5	89.1	76.6	76.9	77.7	85.9	76.8	88.6	76.3	76.2	77.7	76.7	77.0	77.2	80.1	77.1	89.9	80.4	76.2	76.8	89.0	79.4	90.5	89.9	86.1	88.2
3	*Haloarcula amylovorans* LR21^T	78.8	75.7	*	75.3	80.0	78.2	76.8	77.6	75.7	76.8	77.8	88.4	78.7	76.3	79.1	76.4	87.4	77.5	77.7	77.0	79.7	77.6	79.6	79.2	78.6	76.9	77.7	77.6	75.4	79.7	78.2	78.6	76.4	75.8
4	*Haloarcula argentinensis* DSM 12282^T	78.9	89.2	75.3	*	87.4	90.7	76.2	77.0	89.4	90.1	76.1	76.4	77.0	84.6	76.3	89.2	75.7	75.6	76.9	76.2	76.6	76.7	79.3	76.3	89.7	79.7	75.9	76.2	94.4	78.8	90.9	89.5	85.0	87.6
5	*Haloarcula brevis* DT43^T	79.3	88.4	80.0	87.4	*	87.1	79.4	79.9	88.1	87.4	81.0	81.2	80.8	87.5	80.9	87.3	81.1	80.2	80.0	79.6	81.1	79.4	80.1	81.7	87.7	85.8	80.2	87.7	87.4	79.7	86.9	88.1	80.5	87.3
6	*Haloarcula californiae* ATCC 33799^T	77.4	90.3	78.2	90.7	87.1	*	77.5	78.0	91.1	90.0	79.1	79.3	78.3	87.0	79.3	98.2	79.1	78.3	78.0	77.8	79.2	77.9	78.2	79.6	89.3	82.5	78.8	78.8	90.6	77.4	97.8	89.7	87.4	88.9
7	*Haloarcula halophila* DFY41^T	95.5	76.7	76.8	76.2	79.4	77.5	*	77.9	76.6	76.4	78.1	78.3	78.5	76.9	78.1	76.2	77.4	77.8	77.8	85.2	78.4	77.5	79.2	78.4	78.0	77.7	77.7	78.0	76.1	78.6	77.7	78.0	77.4	76.8
8	*Haloarcula halobia* XH51^T	79.0	77.7	77.6	77.0	79.9	78.0	77.9	*	77.6	77.3	78.7	78.8	79.1	77.7	78.9	77.0	77.9	78.4	85.7	77.8	79.4	78.1	92.5	79.1	78.8	78.9	78.5	78.6	77.0	79.0	78.3	78.6	77.7	77.3
9	*Haloarcula hispanica* ATCC 33960^T	79.3	94.5	75.7	89.4	88.1	91.1	76.6	77.6	*	89.0	76.7	76.8	77.7	85.7	76.9	89.4	76.1	76.1	77.4	76.8	77.0	77.0	79.9	77.0	89.8	80.4	76.3	76.7	89.2	78.9	91.0	90.1	86.0	88.1
10	*Haloarcula japonica* DSM 6131^T	79.1	89.1	76.8	90.1	87.4	90.0	76.4	77.3	89.0	*	76.8	76.6	77.5	85.7	76.6	88.7	76.1	76.1	77.1	76.6	76.9	76.8	79.7	77.1	89.5	80.1	76.5	76.4	90.8	78.9	90.0	89.4	85.9	87.3
11	*Haloarcula laminariae* LYG-108^T	79.9	76.6	77.8	76.1	81.0	79.1	78.1	78.7	76.7	76.8	*	77.9	79.5	77.4	78.1	76.4	77.3	83.1	78.6	78.3	78.5	85.1	80.8	78.9	79.9	77.7	83.7	82.4	76.2	90.1	79.0	79.8	77.0	76.3
12	*Haloarcula limicola* YGHS32^T	79.9	76.9	88.4	76.4	81.2	79.3	78.3	78.8	76.8	76.6	77.9	*	80.6	76.9	79.7	76.3	90.0	78.2	78.8	78.7	80.3	79.0	81.5	80.2	80.1	78.5	78.1	78.7	76.4	81.0	79.2	79.8	77.0	76.3
13	*Haloarcula litorea* GDY20^T	79.8	77.7	78.7	77.0	80.8	78.3	78.5	79.1	77.7	77.5	79.5	80.6	*	78.1	79.9	76.9	79.6	79.5	78.7	78.4	80.5	78.8	80.3	80.3	79.0	79.1	79.1	79.5	77.0	80.2	78.2	79.0	78.2	77.4
14	*Haloarcula mannanilytica* MD-130^T	79.0	85.9	76.3	84.6	87.5	87.0	76.9	77.7	85.7	85.7	77.4	76.9	78.1	*	76.8	84.9	76.5	76.7	77.5	76.9	77.2	77.2	80.1	77.5	87.2	80.7	76.9	76.7	84.7	79.3	86.9	87.1	88.5	85.1
15	*Haloarcula marina* DT1^T	79.6	76.8	79.1	76.3	80.9	79.3	78.1	78.9	76.9	76.6	78.1	79.7	79.9	76.8	*	76.2	78.6	78.1	79.0	78.4	80.9	78.9	81.0	80.6	79.8	77.7	77.8	78.4	76.3	80.7	79.2	79.9	76.6	76.2
16	*Haloarcula marismortui* ATCC 43049^T	78.6	88.6	76.4	89.2	87.3	98.2	76.2	77.0	89.4	88.7	76.4	76.3	76.9	84.9	76.2	*	75.8	76.2	76.9	76.3	76.6	76.6	79.0	76.8	89.8	79.6	76.4	76.2	89.0	78.6	98.3	89.8	84.9	87.5
17	*Haloarcula nitratireducens* F27^T	80.3	76.3	87.4	75.7	81.1	79.1	77.4	77.9	76.1	76.1	77.3	90.0	79.6	76.5	78.6	75.8	*	78.3	77.8	77.4	80.4	77.9	81.0	79.9	79.5	77.6	77.9	78.8	75.7	80.8	79.1	79.6	77.1	76.3
18	*Haloarcula onubensis* S3CR25-11^T	79.3	76.2	77.5	75.6	80.2	78.3	77.8	78.4	76.1	76.1	83.1	78.2	79.5	76.7	78.1	76.2	78.3	*	78.2	78.3	78.4	84.7	80.2	79.8	79.3	77.1	84.7	83.6	75.6	86.2	78.5	79.3	77.1	75.9
19	*Haloarcula ordinaria* ZS-22-S1^T	78.9	77.7	77.7	76.9	80.0	78.0	77.8	85.7	77.4	77.1	78.6	78.8	78.7	77.5	79.0	76.9	77.8	78.2	*	78.1	79.3	77.8	86.9	78.9	78.7	78.8	77.9	78.5	77.0	78.9	78.2	78.6	77.6	77.1
20	*Haloarcula pelagica* YJ-61-S^T	86.5	76.7	77.0	76.2	79.6	77.8	85.2	77.8	76.8	76.6	78.3	78.7	78.4	76.9	78.4	76.3	77.4	78.3	78.1	*	78.7	77.1	79.6	78.6	78.5	77.9	78.3	78.4	76.2	78.8	77.6	78.2	77.6	77.0
21	*Haloarcula pellucida* CECT 7537^T	79.9	77.0	79.7	76.6	81.1	79.2	78.4	79.4	77.0	76.9	78.5	80.3	80.5	77.2	80.9	76.6	80.4	78.4	79.3	78.7	*	79.3	81.1	86.8	80.0	78.2	78.4	78.8	76.6	81.1	79.2	79.9	77.1	76.6
22	*Haloarcula rara* SHR3^T	78.5	77.2	77.6	76.7	79.4	77.9	77.5	78.1	77.0	76.8	85.1	79.0	78.8	77.2	78.9	76.6	77.9	84.7	77.8	77.1	79.3	*	79.1	78.6	78.4	77.9	89.8	84.0	76.7	86.4	77.8	78.6	77.1	76.9
23	*Haloarcula regularis* SYNS111^T	78.9	80.1	79.6	79.3	80.1	78.2	79.2	92.5	79.9	79.7	80.8	81.5	80.3	80.1	81.0	79.0	81.0	80.2	86.9	79.6	81.1	79.1	*	81.7	79.0	80.9	80.2	79.8	79.4	79.0	78.4	78.8	80.2	79.7
24	*Haloarcula rubra* F13^T	80.3	77.1	79.2	76.3	81.7	79.6	78.4	79.1	77.0	77.1	78.9	80.2	80.3	77.5	80.6	76.8	79.9	79.8	78.9	78.6	86.8	78.6	81.7	*	80.3	78.0	79.3	80.0	76.5	81.2	79.6	80.4	78.3	76.9
25	*Haloarcula rubripromontorii* SL3^T	78.1	89.9	78.6	89.7	87.7	89.3	78.0	78.8	89.8	89.5	79.9	80.1	79.0	87.2	79.8	89.8	79.5	79.3	78.7	78.5	80.0	78.4	79.0	80.3	*	83.4	79.2	79.5	89.7	78.3	89.4	92.8	87.3	90.3
26	*Haloarcula salina* YGHS18^T	80.0	80.4	76.9	79.7	85.8	82.5	77.7	78.9	80.4	80.1	77.7	78.5	79.1	80.7	77.7	79.6	77.6	77.1	78.8	77.9	78.2	77.9	80.9	78.0	83.4	*	77.1	77.5	79.7	80.1	82.4	83.3	80.7	79.9
27	*Haloarcula salinisoli* F24A^T	79.4	76.2	77.7	75.9	80.2	78.8	77.7	78.5	76.3	76.5	83.7	78.1	79.1	76.9	77.8	76.4	77.9	84.7	77.9	78.3	78.4	89.8	80.2	79.3	79.2	77.1	*	83.2	76.0	86.4	78.6	79.3	76.2	75.9
28	*Haloarcula saliterrae* S1CR25-12^T	79.2	76.8	77.6	76.2	87.7	78.8	78.0	78.6	76.7	76.4	82.4	78.7	79.5	76.7	78.4	76.2	78.8	83.6	78.5	78.4	78.8	84.0	79.8	80.0	79.5	77.5	83.2	*	76.2	79.5	78.7	79.5	77.2	76.1
29	*Haloarcula sebkhae* JCM 19018^T	78.9	89.0	75.4	94.4	87.4	90.6	76.1	77.0	89.2	90.8	76.2	76.4	77.0	84.7	76.3	89.0	75.7	75.6	77.0	76.2	76.6	76.7	79.4	76.5	89.7	79.7	76.0	76.2	*	78.6	90.6	89.7	84.9	87.5
30	*Haloarcula sediminis* CK38^T	78.5	79.4	79.7	78.8	79.7	77.4	78.6	79.0	78.9	78.9	90.1	81.0	80.2	79.3	80.7	78.6	80.8	86.2	78.9	78.8	81.1	86.4	79.0	81.2	78.3	80.1	86.4	79.5	78.6	*	77.5	78.2	85.2	78.8
31	*Haloarcula sinaiiensis* ATCC 33800^T	77.5	90.5	78.2	90.9	86.9	97.8	77.7	78.3	91.0	90.0	79.0	79.2	78.2	86.9	79.2	98.3	79.1	78.5	78.2	77.6	79.2	77.8	78.4	79.6	89.4	82.4	78.6	78.7	90.6	77.5	*	89.7	87.3	88.9
32	*Haloarcula taiwanensis*	78.0	89.9	78.6	89.5	88.1	89.7	78.0	78.6	90.1	89.4	79.8	79.8	79.0	87.1	79.9	89.8	79.6	79.3	78.6	78.2	79.9	78.6	78.8	80.4	92.8	83.3	79.3	79.5	89.7	78.2	89.7	*	87.5	89.8
33	*Haloarcula terrestris* S1AR25-5A^T	79.7	86.1	76.4	85.0	80.5	87.4	77.4	77.7	86.0	85.9	77.0	77.0	78.2	88.5	76.6	84.9	77.1	77.1	77.6	77.6	77.1	77.1	80.2	78.3	87.3	80.7	76.2	77.2	84.9	85.2	87.3	87.5	*	84.9
34	*Haloarcula vallismortis* ATCC 29715^T	78.7	88.2	75.8	87.6	87.3	88.9	76.8	77.3	88.1	87.3	76.3	76.3	77.4	85.1	76.2	87.5	76.3	75.9	77.1	77.0	76.6	76.9	79.7	76.9	90.3	79.9	75.9	76.1	87.5	78.8	88.9	89.8	84.9	*

Table S4. Pairwise AAI values among strain GH36^{T} and the current species of the genus *Haloarcula*. Values exceeding the species-level threshold of 95% are highlighted in yellow.

No.	AAI (%)	1	2	3	4	5	6	7	8	9	10	11	12	13	14	15	16	17	18	19	20	21	22	23	24	25	26	27	28	29	30	31	32	33	34
1	**Strain GH36^{T}**	*	71.0	72.2	71.6	73.0	71.1	94.9	71.9	71.7	71.5	71.5	72.9	72.8	71.9	73.8	71.3	72.2	70.6	72.4	84.9	73.4	71.7	71.8	73.3	71.1	72.0	70.4	70.9	71.2	71.8	71.0	71.6	71.9	71.3
2	***Haloarcula amylolytica* JCM 13557^{T}**	71.0	*	70.9	92.1	87.1	91.2	71.0	71.6	95.9	92.9	70.2	71.7	71.4	88.1	72.5	91.7	71.2	70.0	71.8	70.6	73.2	71.0	71.3	72.5	90.8	79.7	70.1	70.4	91.7	70.7	91.4	90.9	88.3	90.7
3	***Haloarcula amylovorans* LR21^{T}**	72.2	70.9	*	71.0	72.1	71.1	71.8	71.7	71.6	73.1	73.0	90.1	75.1	72.5	77.0	72.8	89.6	72.9	71.9	71.6	77.8	72.4	71.9	76.9	71.4	72.9	72.2	72.7	71.3	72.5	70.8	71.8	72.1	71.6
4	***Haloarcula argentinensis* DSM 12282^{T}**	71.6	92.1	71.0	*	86.8	92.0	71.3	71.6	92.5	92.6	70.8	72.2	71.4	87.4	72.8	92.1	71.4	70.3	71.5	70.8	73.8	70.9	71.3	72.8	90.9	79.6	70.7	70.8	96.1	71.0	92.1	91.2	87.7	90.8
5	***Haloarcula brevis* DT43^{T}**	73.0	87.1	72.1	86.8	*	86.8	72.5	72.8	87.5	87.3	72.2	73.2	72.9	86.6	74.0	86.9	72.4	71.5	72.7	72.3	74.4	72.3	72.6	74.4	87.1	81.5	71.3	72.1	86.9	72.6	87.1	87.8	86.6	86.7
6	***Haloarcula californiae* ATCC 33799^{T}**	71.1	91.2	71.1	92.0	86.8	*	71.3	71.6	91.9	90.9	70.2	71.9	71.3	87.1	72.7	97.7	71.1	69.8	71.4	70.8	73.5	71.7	71.2	72.6	90.4	79.3	70.1	70.7	91.5	71.1	97.3	91.2	87.5	90.5
7	***Haloarcula halophila* DFY41^{T}**	94.9	71.0	71.8	71.3	72.5	71.3	*	71.8	71.3	71.4	71.4	72.7	73.0	71.8	73.7	71.0	71.9	70.9	71.8	85.2	73.5	71.3	71.8	73.2	71.3	72.1	70.3	71.0	70.9	71.7	71.4	71.8	71.9	71.3
8	***Haloarcula halobia* XH51^{T}**	71.9	71.6	71.7	71.6	72.8	71.6	71.8	*	72.1	71.9	70.6	72.8	72.6	72.2	73.9	71.6	72.0	71.5	71.6	72.3	73.9	71.2	91.0	73.6	72.0	72.7	71.4	71.6	71.6	71.5	71.6	72.3	72.1	71.8
9	***Haloarcula hispanica* ATCC 33960^{T}**	71.7	95.9	71.6	92.5	87.5	91.9	71.3	72.1	*	92.0	71.0	72.3	72.1	88.2	73.0	92.4	71.6	70.5	71.8	71.2	74.1	71.3	71.7	73.3	91.1	80.1	70.8	70.9	92.4	71.6	92.1	91.4	88.5	91.1
10	***Haloarcula japonica* DSM 6131^{T}**	71.5	92.9	73.1	92.6	87.3	90.9	71.4	71.9	92.0	*	71.8	72.3	72.1	88.8	72.9	91.8	72.1	70.7	71.7	70.9	73.6	71.1	71.6	73.4	90.0	80.0	71.2	70.9	93.1	71.0	91.2	90.5	88.3	90.4
11	***Haloarcula laminariae* LYG-108^{T}**	71.5	70.2	73.0	70.8	72.2	70.2	71.4	70.6	71.0	71.8	*	73.2	73.1	72.5	74.7	71.2	72.7	81.8	71.6	71.6	74.7	83.3	71.5	75.0	71.0	71.4	82.9	81.1	70.6	87.8	70.6	71.1	72.2	71.3
12	***Haloarcula limicola* YGHS32^{T}**	72.9	71.7	90.1	72.2	73.2	71.9	72.7	72.8	72.3	72.3	73.2	*	76.1	72.8	77.6	72.0	92.6	73.6	72.9	72.6	78.6	73.7	72.9	78.7	72.0	74.1	73.1	74.1	72.0	73.5	71.5	72.4	72.6	71.9
13	***Haloarcula litorea* GDY20^{T}**	72.8	71.4	75.1	71.4	72.9	71.3	73.0	72.6	72.1	72.1	73.1	76.1	*	72.9	76.0	71.8	75.8	73.7	72.3	73.0	76.5	72.5	72.7	77.2	71.7	72.8	72.4	72.7	71.5	72.6	71.0	71.7	72.1	71.6
14	***Haloarcula mannanilytica* MD-130^{T}**	71.9	88.1	72.5	87.4	86.6	87.1	71.8	72.2	88.2	88.8	72.5	72.8	72.9	*	73.2	88.0	72.4	71.8	71.7	71.5	73.6	71.1	72.5	74.0	87.3	81.0	71.5	71.3	87.4	71.5	87.2	87.1	91.7	87.6
15	***Haloarcula marina* DT1^{T}**	73.8	72.5	77.0	72.8	74.0	72.7	73.7	73.9	73.0	72.9	74.7	77.6	76.0	73.2	*	73.0	77.2	74.9	74.4	73.5	80.5	74.7	74.1	80.3	72.5	73.7	74.4	75.3	72.5	75.2	72.7	72.9	73.0	72.7
16	***Haloarcula marismortui* ATCC 43049^{T}**	71.3	91.7	72.8	92.1	86.9	97.7	71.0	71.6	92.4	91.8	71.2	72.0	71.8	88.0	73.0	*	71.9	71.0	71.4	70.9	73.7	71.3	71.7	73.4	91.0	79.4	70.9	71.2	91.8	70.9	97.7	91.5	87.6	91.1
17	***Haloarcula nitratireducens* F27^{T}**	72.2	71.2	89.6	71.4	72.4	71.1	71.9	72.0	71.6	72.1	72.7	92.6	75.8	72.4	77.2	71.9	*	73.4	72.2	71.7	77.9	72.7	72.5	77.8	71.4	73.3	72.4	73.3	71.4	72.6	71.0	71.8	72.3	71.4
18	***Haloarcula onubensis* S3CR25-11^{T}**	70.6	70.0	72.9	70.3	71.5	69.8	70.9	71.5	70.5	70.7	81.8	73.6	73.7	71.8	74.9	71.0	73.4	*	71.6	71.1	74.5	84.1	71.5	75.8	70.3	71.1	84.0	82.2	70.2	82.5	69.9	70.5	71.7	70.7
19	***Haloarcula ordinaria* ZS-22-S1^{T}**	72.4	71.8	71.9	71.5	72.7	71.4	71.8	71.6	71.8	71.7	71.6	72.9	72.3	71.7	74.4	71.4	72.2	71.6	*	72.5	74.0	71.1	84.1	74.3	71.5	72.8	71.7	71.8	71.6	71.9	71.7	71.8	72.1	71.3
20	***Haloarcula pelagica* YJ-61-S^{T}**	84.9	70.6	71.6	70.8	72.3	70.8	85.2	72.3	71.2	70.9	71.6	72.6	73.0	71.5	73.5	70.9	71.7	71.1	72.5	*	73.4	71.0	71.9	73.3	70.6	72.6	70.6	71.3	70.6	71.1	70.8	71.3	71.2	70.8
21	***Haloarcula pellucida* CECT 7537^{T}**	73.4	73.2	77.8	73.8	74.4	73.5	73.5	73.9	74.1	73.6	74.7	78.6	76.5	73.6	80.5	73.7	77.9	74.5	74.0	73.4	*	74.7	74.0	88.5	73.8	74.5	74.8	75.3	73.5	75.2	73.4	91.1	73.6	73.7
22	***Haloarcula rara* SHR3^{T}**	71.7	71.0	72.4	70.9	72.3	71.7	71.3	71.2	71.3	71.1	83.3	73.7	72.5	71.1	74.7	71.3	72.7	84.1	71.1	71.0	74.7	*	71.4	74.3	71.8	71.8	89.7	83.0	70.8	84.5	71.6	72.0	71.9	71.1
23	***Haloarcula regularis* SYNS111^{T}**	71.8	71.3	71.9	71.3	72.6	71.2	71.8	91.0	71.7	71.6	71.5	72.9	72.7	72.5	74.1	71.7	72.5	71.5	84.1	71.9	74.0	71.4	*	74.2	71.4	72.6	70.7	71.3	71.3	71.2	71.4	71.7	71.8	71.7
24	***Haloarcula rubra* F13^{T}**	73.3	72.5	76.9	72.8	74.4	72.6	73.2	73.6	73.3	73.4	75.0	78.7	77.2	74.0	80.3	73.4	77.8	75.8	74.3	73.3	88.5	74.3	74.2	*	72.8	74.3	75.1	75.7	72.4	73.9	72.5	73.3	74.5	72.9
25	***Haloarcula rubripromontorii* SL3^{T}**	71.1	90.8	71.4	90.9	87.1	90.4	71.3	72.0	91.1	90.0	71.0	72.0	71.7	87.3	72.5	91.0	71.4	70.3	71.5	70.6	73.8	71.8	71.4	72.8	*	79.5	70.5	71.3	90.9	71.5	90.6	94.0	87.2	91.4
26	***Haloarcula salina* YGHS18^{T}**	72.0	79.7	72.9	79.6	81.5	79.3	72.1	72.7	80.1	80.0	71.4	74.1	72.8	81.0	73.7	79.4	73.3	71.1	72.8	72.6	74.5	71.8	72.6	74.3	79.5	*	71.2	71.5	79.6	71.5	79.1	80.0	80.4	79.6
27	***Haloarcula salinisoli* F24A^{T}**	70.4	70.1	72.2	70.7	71.3	70.1	70.3	71.4	70.8	71.2	82.9	73.1	72.4	71.5	74.4	70.9	72.4	84.0	71.7	70.6	74.8	89.7	70.7	75.1	70.5	71.2	*	82.5	70.4	83.2	70.1	70.9	71.1	70.8
28	***Haloarcula saliterrae* S1CR25-12^{T}**	70.9	70.4	72.7	70.8	72.1	70.7	71.0	71.6	70.9	70.9	81.1	74.1	72.7	71.3	75.3	71.2	73.3	82.2	71.8	71.3	75.3	83.0	71.3	75.7	71.3	71.5	82.5	*	70.6	81.4	70.6	71.3	71.5	71.1
29	***Haloarcula sebkhae* JCM 19018^{T}**	71.2	91.7	71.3	96.1	86.9	91.5	70.9	71.6	92.4	93.1	70.6	72.0	71.5	87.4	72.5	91.8	71.4	70.2	71.6	70.6	73.5	70.8	71.3	72.4	90.9	79.6	70.4	70.6	*	70.9	91.7	90.8	70.6	90.6
30	***Haloarcula sediminis* CK38^{T}**	71.8	70.7	72.5	71.0	72.6	71.1	71.7	71.5	71.6	71.0	87.8	73.5	72.6	71.5	75.2	70.9	72.6	82.5	71.9	71.1	75.2	84.5	71.2	73.9	71.5	71.5	83.2	81.4	70.9	*	71.1	71.3	71.5	71.3
31	***Haloarcula sinaiiensis* ATCC 33800^{T}**	71.0	91.4	70.8	92.1	87.1	97.3	71.4	71.6	92.1	91.2	70.6	71.5	71.0	87.2	72.7	97.7	71.0	69.9	71.7	70.8	73.4	71.6	71.4	72.5	90.6	79.1	70.1	70.6	91.7	71.1	*	91.6	87.3	90.7
32	***Haloarcula taiwanensis***	71.6	90.9	71.8	91.2	87.8	91.2	71.8	72.3	91.4	90.5	71.1	72.4	71.7	87.1	72.9	91.5	71.8	70.5	71.8	71.3	91.1	72.0	71.7	73.3	94.0	80.0	70.9	71.3	90.8	71.3	91.6	*	87.6	91.0
33	***Haloarcula terrestris* S1AR25-5A^{T}**	71.9	88.3	72.1	87.7	86.6	87.5	71.9	72.1	88.5	88.3	72.2	72.6	72.1	91.7	73.0	87.6	72.3	71.7	72.1	71.2	73.6	71.9	71.8	74.5	87.2	80.4	71.1	71.5	70.6	71.5	87.3	87.6	*	87.4
34	***Haloarcula vallismortis* ATCC 29715^{T}**	71.3	90.7	71.6	90.8	86.7	90.5	71.3	71.8	91.1	90.4	71.3	71.9	71.6	87.6	72.7	91.1	71.4	70.7	71.3	70.8	73.7	71.1	71.7	72.9	91.4	79.6	70.8	71.1	90.6	71.3	90.7	91.0	87.4	*

Table S5. Pairwise dDDH values among strain GH36^T and the current species of the genus *Haloarcula*. Values exceeding the species-level threshold of 70% are highlighted in yellow.

No.	dDDH (%)	1	2	3	4	5	6	7	8	9	10	11	12	13	14	15	16	17	18	19	20	21	22	23	24	25	26	27	28	29	30	31	32	33	34
1	Strain GH36^T	*	26.0	25.8	25.2	26.9	26.1	67.1	26.5	26.1	26.2	26.9	26.7	27.2	25.9	26.3	25.9	26.8	26.0	26.7	35.6	26.7	26.1	27.0	27.1	26.4	26.9	26.2	26.5	25.8	27.0	26.3	26.3	26.4	26.1
2	*Haloarcula amylolytica* JCM 13557^T	26.0	*	21.8	39.0	37.8	42.7	26.7	26.6	58.9	39.9	22.3	22.3	26.5	32.9	22.1	38.1	22.3	22.3	26.9	26.3	22.0	26.1	26.7	22.7	40.6	25.0	22.1	22.2	39.0	26.7	42.8	41.3	33.8	36.0
3	*Haloarcula amylovorans* LR21^T	25.8	21.8	*	21.4	26.5	25.6	25.8	26.5	21.5	23.7	23.1	36.8	27.4	22.5	23.1	23.6	37.2	23.1	26.7	26.3	23.7	26.6	26.7	24.3	25.9	22.0	23.4	22.7	21.3	26.6	25.4	25.7	21.9	21.5
4	*Haloarcula argentinensis* DSM 12282^T	25.2	39.0	21.4	*	35.9	43.9	25.9	25.3	39.2	42.7	21.7	21.7	25.9	30.6	21.6	39.7	21.8	21.8	25.8	25.8	21.8	25.8	25.6	22.0	40.7	24.0	21.9	22.2	60.0	26.0	43.8	40.3	31.0	34.6
5	*Haloarcula brevis* DT43^T	26.9	37.8	26.5	35.9	*	36.0	26.9	26.8	37.5	36.7	27.4	27.3	27.3	36.4	27.3	35.6	27.6	26.8	27.2	27.2	27.3	26.5	27.4	27.9	38.0	34.1	26.6	36.7	36.3	27.2	35.8	38.8	27.0	36.3
6	*Haloarcula californiae* ATCC 33799^T	26.1	42.7	25.6	43.9	36.0	*	26.0	25.4	44.8	41.9	26.1	25.9	26.0	35.7	26.0	85.7	25.8	25.1	25.8	26.3	26.1	25.5	26.1	26.7	40.4	29.6	25.7	25.7	43.3	26.3	85.0	40.7	36.5	39.0
7	*Haloarcula halophila* DFY41^T	67.1	26.7	25.8	25.9	26.9	26.0	*	26.6	26.1	26.2	27.1	27.0	27.5	26.4	26.1	25.9	26.8	26.6	26.6	35.7	26.7	26.2	27.2	27.5	26.4	27.0	26.3	26.6	26.1	27.0	26.3	26.3	26.3	26.2
8	*Haloarcula halobia* XH51^T	26.5	26.6	26.5	25.3	26.8	25.4	26.6	*	26.1	26.3	26.7	27.7	27.7	26.2	27.4	25.6	27.7	26.8	36.0	26.9	27.6	26.1	51.6	27.7	25.9	27.7	26.7	26.7	25.6	26.6	25.7	26.1	26.0	25.5
9	*Haloarcula hispanica* ATCC 33960^T	26.1	58.9	21.5	39.2	37.5	44.8	26.1	26.1	*	39.7	22.3	22.3	26.3	32.5	22.0	40.4	22.1	22.3	26.1	26.4	22.0	26.0	26.3	22.4	40.7	25.0	22.0	22.3	39.1	26.2	44.8	41.4	33.3	35.9
10	*Haloarcula japonica* DSM 6131^T	26.2	39.9	23.7	42.7	36.7	41.9	26.2	26.3	39.7	*	23.1	22.0	26.5	32.1	21.9	38.3	22.3	23.0	26.0	26.0	21.9	25.9	26.6	22.7	40.4	25.0	23.0	22.1	44.0	26.6	42.0	40.2	32.2	34.5
11	*Haloarcula laminariae* LYG-108^T	26.9	22.3	23.1	21.7	27.4	26.1	27.1	26.7	22.3	23.1	*	23.2	27.8	23.0	23.1	22.8	23.7	29.1	27.5	27.1	23.2	34.8	27.3	24.7	26.5	23.0	29.7	27.7	21.9	42.6	26.0	26.6	23.1	22.7
12	*Haloarcula limicola* YGHS32^T	26.7	22.3	36.8	21.7	27.3	25.9	27.0	27.7	22.3	22.0	23.2	*	28.7	21.9	23.6	21.9	46.6	23.1	27.6	27.1	24.2	27.7	27.8	24.8	26.4	23.0	22.9	23.2	21.9	27.6	25.9	26.6	22.1	21.8
13	*Haloarcula litorea* GDY20^T	27.2	26.5	27.4	25.9	27.3	26.0	27.5	27.7	26.3	26.5	27.8	28.7	*	26.9	27.9	26.4	28.8	28.1	27.1	27.8	28.2	27.4	27.9	29.1	26.4	28.0	26.9	27.3	26.1	27.3	25.8	26.4	26.6	26.3
14	*Haloarcula mannanilytica* MD-130^T	25.9	32.9	22.5	30.6	36.4	35.7	26.4	26.2	32.5	32.1	23.0	21.9	26.9	*	21.8	30.9	22.5	23.1	26.0	26.1	21.9	25.6	27.1	22.9	36.4	25.0	23.0	21.9	30.6	26.1	35.7	35.6	38.0	31.1
15	*Haloarcula marina* DT1^T	26.3	22.1	23.1	21.6	27.3	26.0	26.1	27.4	22.0	21.9	23.1	23.6	27.9	21.8	*	22.1	23.7	23.0	27.2	26.4	24.7	27.6	27.7	25.2	26.6	23.0	23.0	23.2	21.5	27.4	26.0	26.5	21.9	21.6
16	*Haloarcula marismortui* ATCC 43049^T	25.9	38.1	23.6	39.7	35.6	85.7	25.9	25.6	40.4	38.3	22.8	21.9	26.4	30.9	22.1	*	22.0	22.7	26.0	26.0	21.7	25.3	26.0	22.6	40.3	24.0	23.1	22.2	38.9	25.6	86.4	40.3	31.0	34.4
17	*Haloarcula nitratireducens* F27^T	26.8	22.3	37.2	21.8	27.6	25.8	26.8	27.7	22.1	22.3	23.7	46.6	28.8	22.5	23.7	22.0	*	23.6	27.6	27.1	24.5	27.5	28.0	25.4	26.4	23.0	23.1	23.7	21.7	27.5	25.7	26.1	22.7	21.7
18	*Haloarcula onubensis* S3CR25-11^T	26.0	22.3	23.1	21.8	26.8	25.1	26.6	26.8	22.3	23.0	29.1	23.1	28.1	23.1	23.0	22.7	23.6	*	26.7	27.2	23.2	34.2	27.3	25.1	25.4	22.5	30.9	28.1	21.6	34.0	25.1	25.9	23.8	22.3
19	*Haloarcula ordinaria* ZS-22-S1^T	26.7	26.9	26.7	25.8	27.2	25.8	26.6	36.0	26.1	26.0	27.5	27.6	27.1	26.0	27.2	26.0	27.6	26.7	*	28.1	27.4	26.7	36.2	29.1	26.3	27.4	27.8	26.9	26.1	26.8	26.2	26.2	26.5	26.2
20	*Haloarcula pelagica* YJ-61-S^T	35.6	26.3	26.3	25.8	27.2	26.3	35.7	26.9	26.4	26.0	27.1	27.1	27.8	26.1	26.4	26.0	27.1	27.2	28.1	*	26.9	26.4	27.5	27.7	26.6	27.2	27.2	27.6	25.9	26.9	26.1	26.6	26.3	25.8
21	*Haloarcula pellucida* CECT 7537^T	26.7	22.0	23.7	21.8	27.3	26.1	26.7	27.6	22.0	21.9	23.2	24.2	28.2	21.9	24.7	21.7	24.5	23.2	27.4	26.9	*	27.7	27.8	36.3	26.3	23.0	23.1	23.3	21.7	27.7	25.9	27.0	21.8	21.8
22	*Haloarcula rara* SHR3^T	26.1	26.1	26.6	25.8	26.5	25.5	26.2	26.1	26.0	25.9	34.8	27.7	27.4	25.6	27.6	25.3	27.5	34.2	26.7	26.4	27.7	*	26.8	28.4	25.7	26.5	46.0	32.8	25.7	34.9	25.4	26.0	25.6	25.9
23	*Haloarcula regularis* SYNS111^T	27.0	26.7	26.7	25.6	27.4	26.1	27.2	51.6	26.3	26.6	27.3	27.8	27.9	27.1	27.7	26.0	28.0	27.3	36.2	27.5	27.8	26.8	*	28.2	26.2	27.9	26.7	26.9	25.6	27.1	26.2	26.2	26.9	26.2
24	*Haloarcula rubra* F13^T	27.1	22.7	24.3	22.0	27.9	26.7	27.5	27.7	22.4	22.7	24.7	24.8	29.1	22.9	25.2	22.6	25.4	25.1	29.1	27.7	36.3	28.4	28.2	*	26.8	23.0	24.7	25.1	22.0	27.9	27.1	27.1	23.6	22.2
25	*Haloarcula rubripromontorii* SL3^T	26.4	40.6	25.9	40.7	38.0	40.4	26.4	25.9	40.7	40.4	26.5	26.4	26.4	36.4	26.6	40.3	26.4	25.4	26.3	26.6	26.3	25.7	26.2	26.8	*	31.1	25.7	26.1	40.8	26.4	40.1	52.2	36.2	41.8
26	*Haloarcula salina* YGHS18^T	26.9	25.0	22.0	24.0	34.1	29.6	27.0	27.7	25.0	25.0	23.0	23.0	28.0	25.0	23.0	24.0	23.0	22.5	27.4	27.2	23.0	26.5	27.9	23.0	31.1	*	23.0	22.5	24.3	26.7	29.5	31.2	25.0	24.3
27	*Haloarcula salinisoli* F24A^T	26.2	22.1	23.4	21.9	26.6	25.7	26.3	26.7	22.0	23.0	29.7	22.9	26.9	23.0	23.0	23.1	23.1	30.9	27.8	27.2	23.1	46.0	26.7	24.7	25.7	23.0	*	28.2	21.9	34.6	25.6	25.9	22.3	22.0
28	*Haloarcula saliterrae* S1CR25-12^T	26.5	22.2	22.7	22.2	36.7	25.7	26.6	26.7	22.3	22.1	27.7	23.2	27.3	21.9	23.2	22.2	23.7	28.1	26.9	27.6	23.3	32.8	26.9	25.1	26.1	22.5	28.2	*	21.8	26.2	25.7	26.3	22.8	22.2
29	*Haloarcula sebkhae* JCM 19018^T	25.8	39.0	21.3	60.0	36.3	43.3	26.1	25.6	39.1	44.0	21.9	21.9	26.1	30.6	21.5	38.9	21.7	21.6	26.1	25.9	21.7	25.7	25.6	22.0	40.8	24.3	21.9	21.8	*	26.1	43.3	40.5	31.2	34.7
30	*Haloarcula sediminis* CK38^T	27.0	26.7	26.6	26.0	27.2	26.3	27.0	26.6	26.2	26.6	42.6	27.6	27.3	26.1	27.4	25.6	27.5	34.0	26.8	26.9	27.7	34.9	27.1	27.9	26.4	26.7	34.6	26.2	26.1	*	26.0	26.5	32.7	26.2
31	*Haloarcula sinaiiensis* ATCC 33800^T	26.3	42.8	25.4	43.8	35.8	85.0	26.3	25.7	44.8	42.0	26.0	25.9	25.8	35.7	26.0	86.4	25.7	25.1	26.2	26.1	25.9	25.4	26.2	27.1	40.1	29.5	25.6	25.7	43.3	26.0	*	40.6	36.3	39.1
32	*Haloarcula taiwanensis*	26.3	41.3	25.7	40.3	38.8	40.7	26.3	26.1	41.4	40.2	26.6	26.6	26.4	35.6	26.5	40.3	26.1	25.9	26.2	26.6	27.0	26.0	26.2	27.1	52.2	31.2	25.9	26.3	40.5	26.5	40.6	*	36.2	40.6
33	*Haloarcula terrestris* S1AR25-5A^T	26.4	33.8	21.9	31.0	27.0	36.5	26.3	26.0	33.3	32.2	23.1	22.1	26.6	38.0	21.9	31.0	22.7	23.8	26.5	26.3	21.8	25.6	26.9	23.6	36.2	25.0	22.3	22.8	31.2	32.7	36.3	36.2	*	30.7
34	*Haloarcula vallismortis* ATCC 29715^T	26.1	36.0	21.5	34.6	36.3	39.0	26.2	25.5	35.9	34.5	22.7	21.8	26.3	31.1	21.6	34.4	21.7	22.3	26.2	25.8	21.8	25.9	26.2	22.2	41.8	24.3	22.0	22.2	34.7	26.2	39.1	40.6	30.7	*

(a)

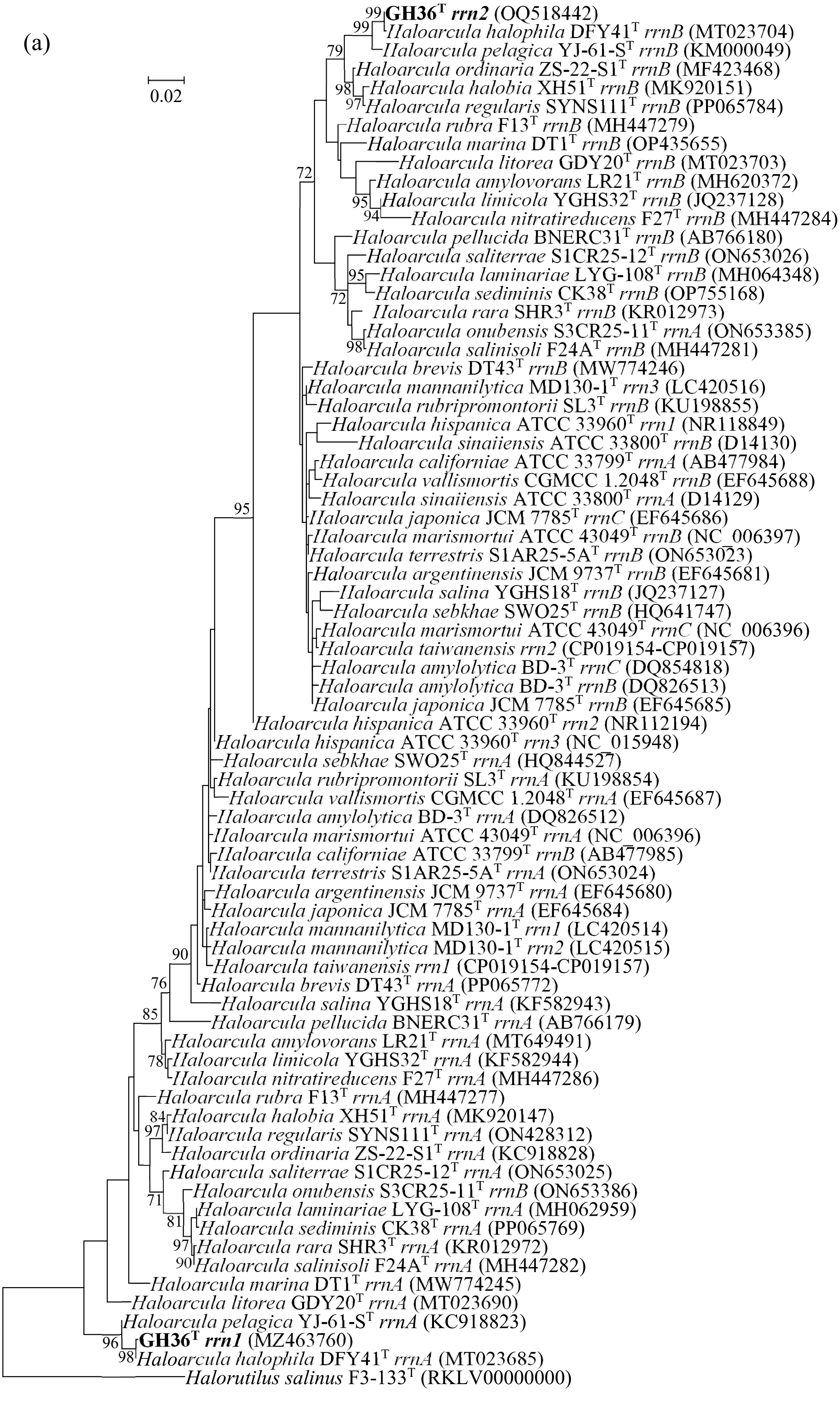

0.02
GH36T rrn2 (OQ518442)
Haloarcula halophila DFY41T rrnB (MT023704)
Haloarcula pelagica YJ-61-ST rrnB (KM000049)
Haloarcula ordinaria ZS-22-S1T rrnB (MF423468)
Haloarcula halobia XH51T rrnB (MK920151)
Haloarcula regularis SYNS111T rrnB (PP065784)
Haloarcula rubra F13T rrnB (MH447279)
Haloarcula marina DT1T rrnB (OP435655)
Haloarcula litorea GDY20T rrnB (MT023703)
Haloarcula amylovorans LR21T rrnB (MH620372)
Haloarcula limicola YGHS32T rrnB (JQ237128)
Haloarcula nitratireducens F27T rrnB (MH447284)
Haloarcula pellucida BNERC31T rrnB (AB766180)
Haloarcula saliterrae S1CR25-12T rrnB (ON653026)
Haloarcula laminariae LYG-108T rrnB (MH064348)
Haloarcula sediminis CK38T rrnB (OP755168)
Haloarcula rara SHR3T rrnB (KR012973)
Haloarcula onubensis S3CR25-11T rrnA (ON653385)
Haloarcula salinisoli F24AT rrnB (MH447281)
Haloarcula brevis DT43T rrnB (MW774246)
Haloarcula mannanilytica MD130-1T rrn3 (LC420516)
Haloarcula rubripromontorii SL3T rrnB (KU198855)
Haloarcula hispanica ATCC 33960T rrn1 (NR118849)
Haloarcula sinaiiensis ATCC 33800T rrnB (D14130)
Haloarcula californiae ATCC 33799T rrnA (AB477984)
Haloarcula vallismortis CGMCC 1.2048T rrnB (EF645688)
Haloarcula sinaiiensis ATCC 33800T rrnA (D14129)
Haloarcula japonica JCM 7785T rrnC (EF645686)
Haloarcula marismortui ATCC 43049T rrnB (NC_006397)
Haloarcula terrestris S1AR25-5AT rrnB (ON653023)
Haloarcula argentinensis JCM 9737T rrnB (EF645681)
Haloarcula salina YGHS18T rrnB (JQ237127)
Haloarcula sebkhae SWO25T rrnB (HQ641747)
Haloarcula marismortui ATCC 43049T rrnC (NC_006396)
Haloarcula taiwanensis rrn2 (CP019154-CP019157)
Haloarcula amylolytica BD-3T rrnC (DQ854818)
Haloarcula amylolytica BD-3T rrnB (DQ826513)
Haloarcula japonica JCM 7785T rrnB (EF645685)
Haloarcula hispanica ATCC 33960T rrn2 (NR112194)
Haloarcula hispanica ATCC 33960T rrn3 (NC_015948)
Haloarcula sebkhae SWO25T rrnA (HQ844527)
Haloarcula rubripromontorii SL3T rrnA (KU198854)
Haloarcula vallismortis CGMCC 1.2048T rrnA (EF645687)
Haloarcula amylolytica BD-3T rrnA (DQ826512)
Haloarcula marismortui ATCC 43049T rrnA (NC_006396)
Haloarcula californiae ATCC 33799T rrnB (AB477985)
Haloarcula terrestris S1AR25-5AT rrnA (ON653024)
Haloarcula argentinensis JCM 9737T rrnA (EF645680)
Haloarcula japonica JCM 7785T rrnA (EF645684)
Haloarcula mannanilytica MD130-1T rrn1 (LC420514)
Haloarcula mannanilytica MD130-1T rrn2 (LC420515)
Haloarcula taiwanensis rrn1 (CP019154-CP019157)
Haloarcula brevis DT43T rrnA (PP065772)
Haloarcula salina YGHS18T rrnA (KF582943)
Haloarcula pellucida BNERC31T rrnA (AB766179)
Haloarcula amylovorans LR21T rrnA (MT649491)
Haloarcula limicola YGHS32T rrnA (KF582944)
Haloarcula nitratireducens F27T rrnA (MH447286)
Haloarcula rubra F13T rrnA (MH447277)
Haloarcula halobia XH51T rrnA (MK920147)
Haloarcula regularis SYNS111T rrnA (ON428312)
Haloarcula ordinaria ZS-22-S1T rrnA (KC918828)
Haloarcula saliterrae S1CR25-12T rrnA (ON653025)
Haloarcula onubensis S3CR25-11T rrnB (ON653386)
Haloarcula laminariae LYG-108T rrnA (MH062959)
Haloarcula sediminis CK38T rrnA (PP065769)
Haloarcula rara SHR3T rrnA (KR012972)
Haloarcula salinisoli F24AT rrnA (MH447282)
Haloarcula marina DT1T rrnA (MW774245)
Haloarcula litorea GDY20T rrnA (MT023690)
Haloarcula pelagica YJ-61-ST rrnA (KC918823)
GH36T rrn1 (MZ463760)
Haloarcula halophila DFY41T rrnA (MT023685)
Halorutilus salinus F3-133T (RKLV00000000)
99
99
79
98
97
72
95
94
95
72
98
95
90
76
85
78
84
97
71
81
97
90
96
98

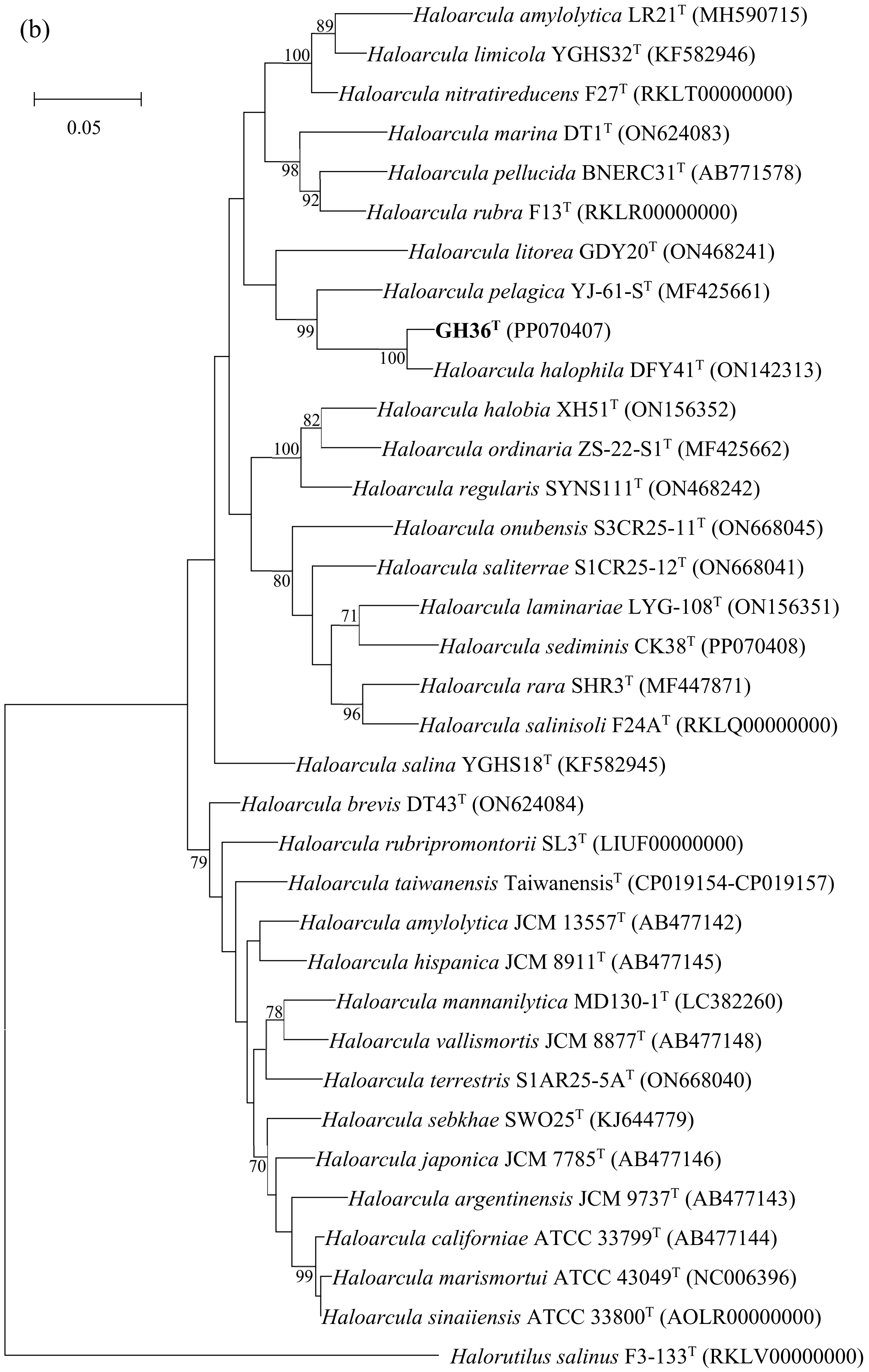

(b)
0.05
Haloarcula amylolytica LR21T (MH590715)
Haloarcula limicola YGHS32T (KF582946)
Haloarcula nitratireducens F27T (RKLT00000000)
Haloarcula marina DT1T (ON624083)
Haloarcula pellucida BNERC31T (AB771578)
Haloarcula rubra F13T (RKLR00000000)
Haloarcula litorea GDY20T (ON468241)
Haloarcula pelagica YJ-61-ST (MF425661)
GH36T (PP070407)
Haloarcula halophila DFY41T (ON142313)
Haloarcula halobia XH51T (ON156352)
Haloarcula ordinaria ZS-22-S1T (MF425662)
Haloarcula regularis SYNS111T (ON468242)
Haloarcula onubensis S3CR25-11T (ON668045)
Haloarcula saliterrae S1CR25-12T (ON668041)
Haloarcula laminariae LYG-108T (ON156351)
Haloarcula sediminis CK38T (PP070408)
Haloarcula rara SHR3T (MF447871)
Haloarcula salinisoli F24AT (RKLQ00000000)
Haloarcula salina YGHS18T (KF582945)
Haloarcula brevis DT43T (ON624084)
Haloarcula rubripromontorii SL3T (LIUF00000000)
Haloarcula taiwanensis TaiwanensisT (CP019154-CP019157)
Haloarcula amylolytica JCM 13557T (AB477142)
Haloarcula hispanica JCM 8911T (AB477145)
Haloarcula mannanilytica MD130-1T (LC382260)
Haloarcula vallismortis JCM 8877T (AB477148)
Haloarcula terrestris S1AR25-5AT (ON668040)
Haloarcula sebkhae SWO25T (KJ644779)
Haloarcula japonica JCM 7785T (AB477146)
Haloarcula argentinensis JCM 9737T (AB477143)
Haloarcula californiae ATCC 33799T (AB477144)
Haloarcula marismortui ATCC 43049T (NC006396)
Haloarcula sinaiiensis ATCC 33800T (AOLR00000000)
Halorutilus salinus F3-133T (RKLV00000000)
89
100
98
92
99
100
82
100
80
71
96
79
78
70
99

Figure S1. Maximum-likelihood phylogenetic trees reconstructed using 16S rRNA (a) and *rpoB'* (b) gene sequences to elucidate the evolutionary relationships among strain GH36^T and the currently recognized species of *Haloarcula*. Bootstrap values, based on 1000 replicates, are displayed for branches with support greater than 70%. Scale bar indicates the expected number of substitutions per nucleotide position.

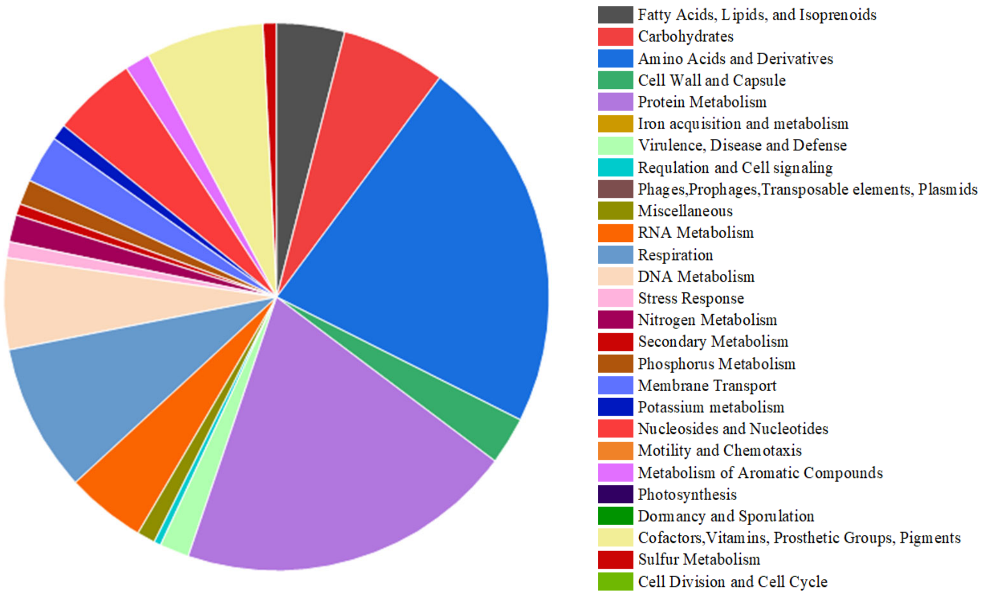

Figure S2. Functional annotation of the genome of strain GH36^T by using the online Rapid Annotation Subsystems Technology (RAST) server.

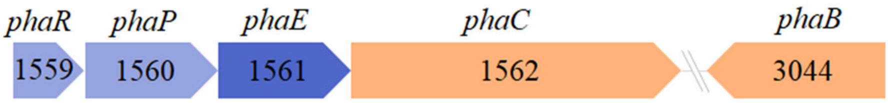

Figure S3. The gene cluster for PHA biosynthesis in strain GH36^{T}. PhaR, PHA synthesis regulatory protein; PhaP, PHA granule binding protein; PhaE and PhaC subunits, type IIIA PHA synthase; PhaB, acetoacetyl CoA reductase. Each gene is represented by a pentagon that indicates its size, location, and transcriptional orientation, with the locus tag value displayed within the pentagon.

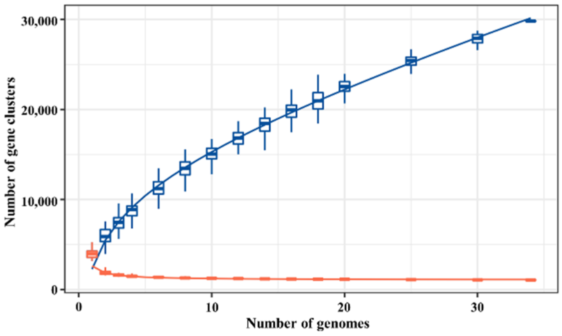

Figure S4. Gene cluster accumulation curve of the pan-genome (blue) and the core-genome (orange) of species of the genus *Haloarcula*.

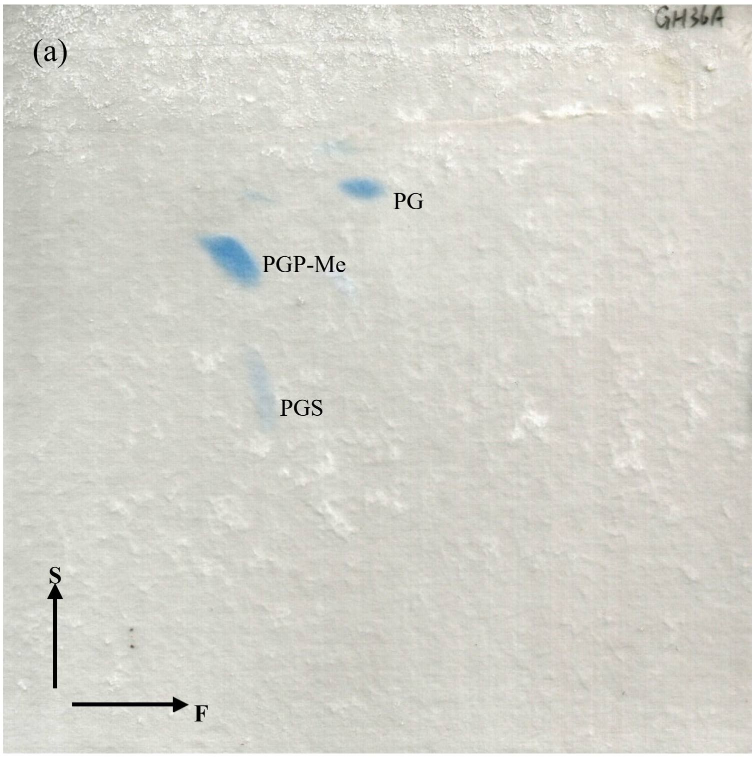
GH36A
(a)
PG
PGP-Me
PGS
S
F

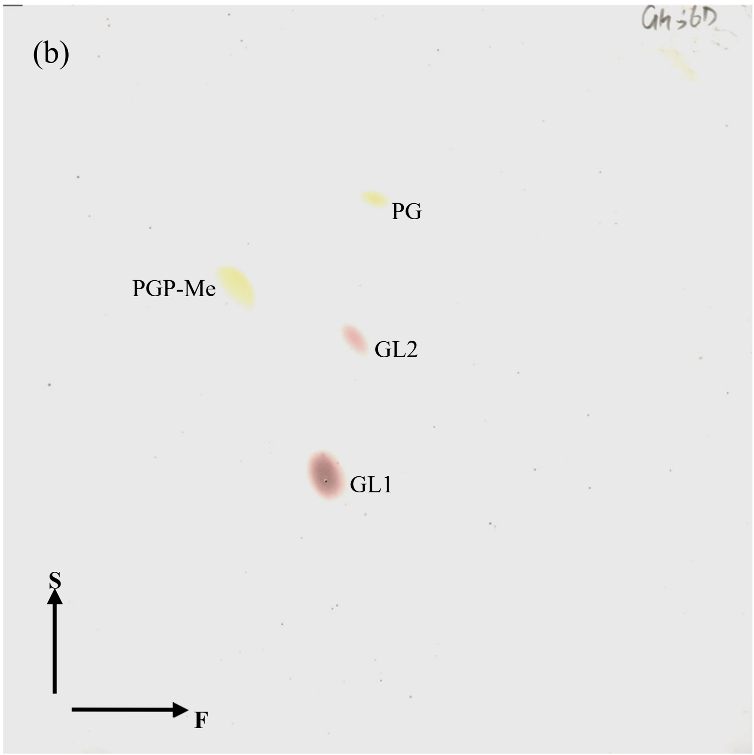
(b)
PG
PGP-Me
GL2
GL1
S
F

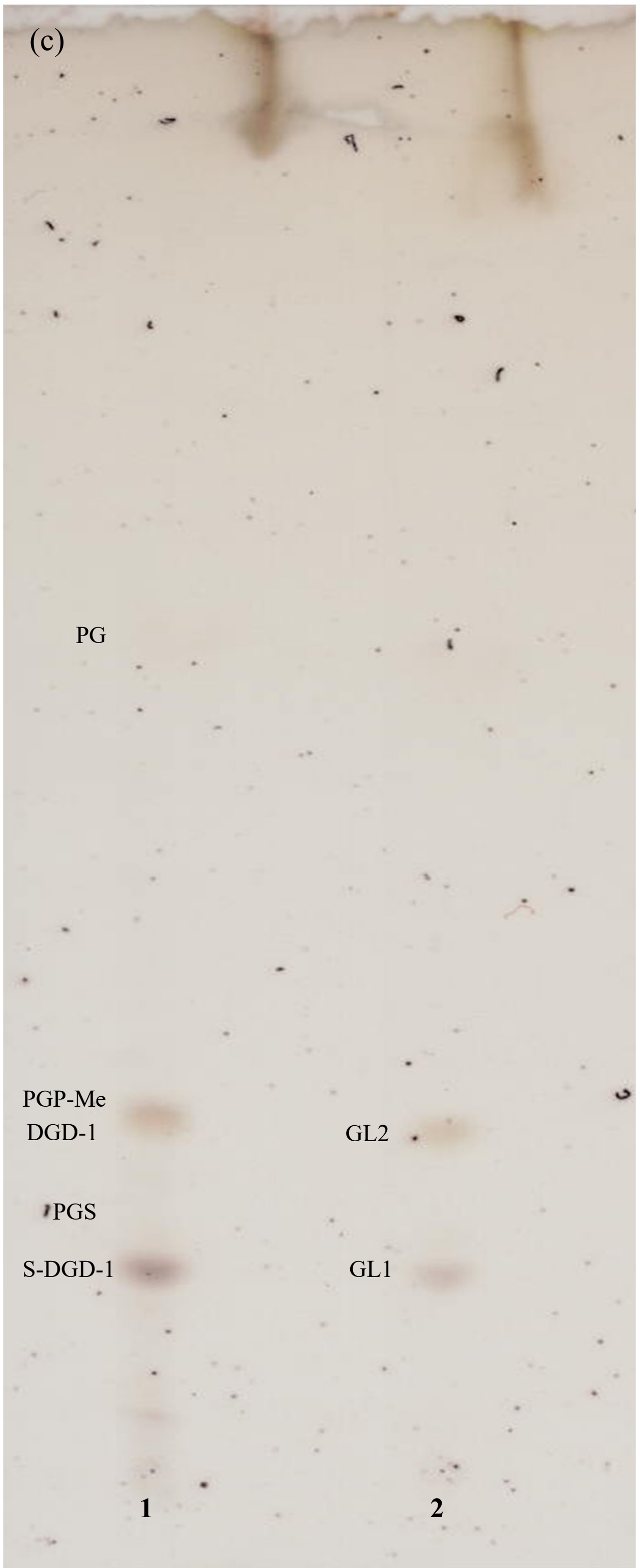

Figure S5. Analysis of the polar lipid composition of strain GH36^T using thin-layer chromatography (TLC). (a) Two-dimensional TLC profile of the phospholipids from strain GH36^T. (b) Two-dimensional TLC profile of the glycolipids and phospholipids from strain GH36^T. (c) One-dimensional TLC profile of the phospholipids and

glycolipids from strain *Haloarcula pellucida* JCM 17820^{T} (lane 1) and strain GH36^{T} (lane 2). Abbreviations: PG, phosphatidylglycerol; PGP-Me, phosphatidylglycerol phosphate methyl ester; PGS, phosphatidylglycerol sulfate; GL, glycolipid; DGD-1, mannosyl glucosyl diether; S-DGD-1, sulfated mannosyl glucosyl diether.
